# Serum fibronectin distinguishes the early stages of hepatocellular carcinoma

**DOI:** 10.1038/s41598-017-09691-3

**Published:** 2017-08-25

**Authors:** Hyunsoo Kim, JiYoung Park, Yongkang Kim, Areum Sohn, Injun Yeo, Su Jong Yu, Jung-Hwan Yoon, Taesung Park, Youngsoo Kim

**Affiliations:** 1 Department of Biomedical Sciences, Seoul National University College of Medicine, Yongon-Dong, Seoul, 110-799 Korea; 2 Department of Biomedical Engineering, Seoul National University College of Medicine, Yongon-Dong, Seoul, 110-799 Korea; 3 Institute of Medical and Biological Engineering, Medical Research Center, Seoul National University College of Medicine, Yongon-Dong, Seoul, 110-799 Korea; 4 Department of Statistics, Seoul National University, Daehak-dong, Seoul, 151-742 Korea; 5 Department of Internal Medicine and Liver Research Institute, Seoul National University Hospital, Yongon-Dong, Seoul, 110-799 Korea

## Abstract

Hepatocellular carcinoma (HCC) is the third leading cause of cancer-related death, necessitating the discovery of serum markers for its early detection. In this study, a total of 180 serum samples from liver cirrhosis (LC), hepatocellular carcinoma (HCC) patients and paired samples of HCC patients who recovered (Recovery) were analyzed by multiple reaction monitoring-mass spectrometry (MRM-MS) to verify biomarkers. The three-fold crossvalidation was repeated 100 times in the training and test sets to evaluate statistical significance of 124 candidate proteins. This step resulted in 2 proteins that had an area under the receiver operating curve (AUROC) values ≥0.800 in the training (n = 90) and test sets (n = 90). Specifically, fibronectin (FN1, WCGTTQNYDADQK), distinguished HCC from LC patients, with an AUROC value of 0.926 by logistic regression. A FN1 protein was selected for validation in an independent sample (n = 60) using enzyme-linked immunosorbent assay (ELISA). The combination of alpha-fetoprotein (AFP) and FN1 improved the diagnostic performance and differentiated HCC patients with normal AFP levels. Our study has examined candidate markers for the benign disease state and malignancy and has followed up on the consequent recovery. Thus, improvement in the early detection of HCC by a 2-marker panel (AFP + FN1) might benefit HCC patients.

## Introduction

Primary liver cancer is one of the most common digestive cancers, of which hepatocellular carcinoma (HCC) accounts for up to 90%^1^. HCC ranks third in annual global cancer mortality rates, and the average time from the discovery of symptoms to death is 6–20 months^2^. Liver cancer usually develops progressively from chronic inflammation, primarily due to hepatitis B and C virus, to liver cirrhosis and ultimately a tumor^3, 4^. The damage and functional impairments to this organ severely affect patient survival^5^. Although preventive strategies for viral infections have stemmed the increase in HCC, liver cancer rates continue to rise in many countries^6, 7^.

Treatment with surgery, chemotherapy, and radiation is effective against the early stages of cancer, necessitating the early detection of liver cancer^8^. Although HCC has been examined extensively^9^ and although its symptoms are well known, its early diagnosis remains difficult; thus, the survival rate after diagnosis is low (<10%)^10^. The conventional diagnostic tools include alpha-fetoprotein (AFP), liver biopsy, and radiographic imaging^11^. As a less invasive and cost-effective procedure, a blood test can be used to measure AFP. Among serological biomarkers, AFP is the approved marker for screening HCC, but it is not used routinely by clinicians, due to its insufficient sensitivity and specificity^12, 13^. To improve the diagnosis and prognosis of HCC, additional reliable markers must be identified that can be used for its early and accurate detection.

Proteomics is considered to be the most powerful tool for the global evaluation of protein expression. Accordingly, proteomic characterization of human serum to discover clinically useful protein biomarkers is promising, because various proteins are expressed and released into the bloodstream in response to specific physiological states^14^. Among the analytical platforms that assess the proteome, liquid chromatography-mass spectrometry that assess the proteome, liquid chromatography-mass spectrometry (LC-MS) is one of the most commonly used approaches for proteomic studies, because it provides good separation and accurate detection of proteins in complex specimens with high sensitivity and resolution^15–18^. Further, multiple reaction monitoring-mass spectrometry (MRM-MS) is widely used for the validation of molecules of interest^19, 20^. This technique can detect attomole levels of specific peptides simultaneously in complex biofluids^21–24^. In addition, the MRM-MS assay generates consistent and reproducible datasets between laboratories in highly complex samples^18^.

In this study, we developed a 2-marker panel [alpha-fetoprotein (AFP) + fibronectin (FN1)] for the early diagnosis of HCC. HCC and liver cirrhosis patient (LC) groups were selected for differentiation between malignant and benign disease status. Recovered patients (Recovery) in whom HCC had regressed but cirrhosis remained were included to ensure the specificity of the markers.

Candidate targets of 929 proteins were analyzed by MRM-MS, and then, the final marker was validated in independent samples using enzyme-linked immunosorbent assay (ELISA). Through logistic regression (LR) analysis, the panel was evaluated in LC versus HCC and HCC versus Recovery groups. The panel also differentiated HCC patients within the normal range of AFP (<20 ng/mL) from LC and recovered patients. Thus, this study increases our understanding of the nature by which HCC develops and can guide clinical practices in diagnosing HCC more easily.

## Results

### Patient population

All patients were matched for characteristics to avoid bias (Table 1). A total of 90 serum samples (30 LC, 30 HCC, and 30 Recovery) were obtained for the training set to select significantly expressed targets. The HCC and Recovery samples were taken from the same patients. The markers were tested in 90 independent serum samples—30 LC, 30 HCC, and 30 Recovery (“Test set”). The final target of the study was re-evaluated in 20 independent LC, HCC, and Recovery samples each by ELISA (“Validation set”). The samples were allocated randomly within the blocked batches. The number of cases and controls were equally arranged and labeled with identification numbers to blind the researchers throughout the depletion, digestion, and MRM-MS analysis.

**Table 1:** Demographic and clinical characteristics of the study population. HBsAg, hepatitis B surface antigen; Anti-HCV, antibody against hepatitis C virus; ECOG, eastern cooperative oncology group; AFP, alpha-fetoprotein; TNM, tumor, node, and metastasis.

		Training set		Test set		Validation set	
		LC	HCC/Recovery	LC	HCC/Recovery	LC	HCC/Recovery
		(*n* = 30)	(*n* = 30/30)	(*n* = 30)	(*n* = 30/30)	(*n* = 20)	(*n* = 20/20)
Age (years)		58.97 ± 8.07	57.83 ± 10.21	55.33 ± 9.77	62.50 ± 9.38	55.00 ± 7.13	57.00 ± 10.17
Gender	Male	19	24	2	21	0	18
	Female	11	6	28	9	20	2
Etiology	HBsAg-positive	21	21	17	21	16	15
	Anti-HCV-positive	1	3	2	4	1	1
	Alcohol	8	6	11	5	3	4
ECOG	0	29	29	30	29	20	20
	1	1	1	0	1	0	0
AFP (ng/mL)	<20	25	21	29	21	20	13
	≥20	5	9	1	9	0	7
Tumor size (cm)	<5		29		27		17
	≥5		1		3		3
Tumor number	<3		30		28		19
	≥3		0		2		1
Metastasis	No		30		30		20
	Yes		0		0		0
TNM stage	0		0		0		0
	I		27		23		17
	II		3		7		3
	III		0		0		0
	IV		0		0		0

### Target candidate selection

From the list of potential markers that was provided by the LiverAtlas database, 50,208 proteins that were related to human liver disease with a reliability score ≥3 were selected^25^. Of these proteins, 1,683 secreted proteins, based on the Uniprot database, were included. Ultimately, 929 proteins (5,544 peptides) with well-matched MS/MS libraries from the National Institute of Standards and Technology (NIST) were analyzed by mass spectrometry^26^. To select candidate protein markers that were detected reproducibly, semiquantitative MRM-MS was performed on a pooled serum sample (60 HCC). The pooled serum sample was analyzed in triplicate to select detectable peptides.

The targets were considered to be detectable if: (i) the transitions of a peptide had a signal-to-noise ratio (S/N) ≥10; (ii) at least 4 light MRM-MS transitions were observed; (iii) the same elution patterns and ratios of transition peaks as in the spectral library were obtained (dot product >0.6)^27^. The dot product score represents the correlation when the peak intensities of transitions that are derived from peptides of interest are compared with the corresponding entries in the spectrum library. The technical reproducibility [coefficient of variation (CV) <20%] was confirmed with triplicate sets of data.

Then, unique peptides were chosen by aligning the target peptide sequences to the corresponding regions of source proteins with the Basic Local Alignment Search Tool (BLAST) from the National Center for Biotechnology Information (NCBI). As a result, 225 proteins and 488 peptides were selected for further verification.

### Interference screen of the peptides and measurements of endogenous peptide concentrations

To screen for interference of endogenous target peptides in subjects, 488 unpurified stable-isotope-labeled standard (SIS) peptides that corresponded to their natural counterparts (endogenous peptides, light) were synthesized with heavy isotope-labeled lysine (^13^C_6_^15^N_2_) or arginine (^13^C_6_^15^N_4_) at their C-termini (SIS peptides, heavy). The unpurified SIS peptide mixture (corresponding to 488 peptides) was prepared (20 nM, 200 nM, and 2,000 nM) and spiked into a pooled serum samples (60 HCC). The pooled serum sample was analyzed in triplicate. The automated detection of inaccurate and imprecise transitions (AuDIT) algorithm was used to identify and eliminate contributions from falsely transmitted ions^28^. Data with *P* > 0.05 in the comparison between the product ion intensity of endogenous peptides and SIS peptides and consistent peak areas, regardless of repeated analysis (CV < 0.2), were selected for further analysis. Of 488 peptides that were surveyed, 231 corresponding to 124 proteins, were verified as interference-free in pooled HCC serum. (Supplementary Table S1).

Endogenous levels were estimated using Skyline with a confidence level of 0.95. SIS peptides were titrated at 3 concentrations (20 nM, 200 nM, and 2000 nM) in a pooled HCC serum to generate a titration curve. Then, the proper spiking amount (0.1< endogenous/SIS peptide <10) of each peptide standards was determined. For example, the SIS peptide mixture was spiked at 200 nM, and the ratio of endogenous to SIS peptide was calculated. Technical replicates 1, 2, and 3 had ratios of 0.80, 0.75, and 0.76, respectively. By multiplying 200 fmol to each ratio, the endogenous levels for each replicate set were estimated to be 160 fmol, 150 fmol, and 152 fmol. An endogenous level of 154 fmol was derived by averaging these 3 values (Supplementary Table S1).

### Diagnostic performance of single marker evaluated by MRM-MS

**Figure 1:**
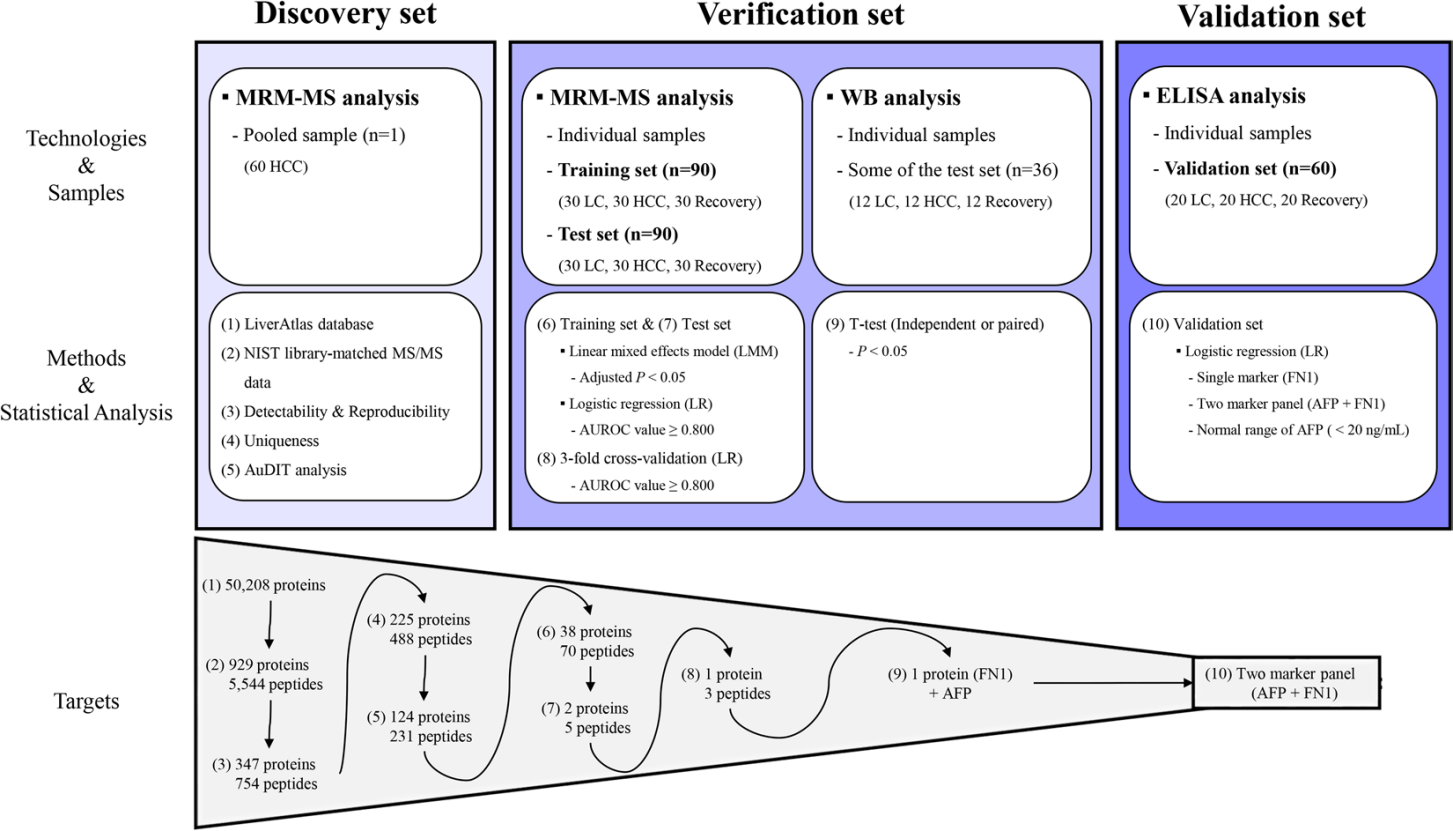
Overall scheme of the study. Reproducibly detected peptides were selected from pooled serum samples of hepatocellular carcinoma patients. In the training set of 30 liver cirrhosis, 30 hepatocellular carcinoma, and 30 recovered patients, significantly differentiated proteins were verified. The single targets with AUROC values  ≥ 0.800 were tested in 90 independent samples, composed of liver cirrhosis, hepatocellular carcinoma, and recovered patients. The final target, FN1, was verified by antibody-based assays, such as western blot. FN1 was combined with AFP into a 2-marker panel which was validated in 60 independent samples by ELISA.

The 231-plex MRM-MS assay was applied to the individual serum samples. Averages of the technical triplicates were used to construct a more precise multi-marker panel in the training set. The performance of the resulting multi-marker panel was evaluated by analyzing the test set samples with a single measurement (Supplementary Figs S1 and S2). We obtained the relative abundance (fold-change and adjusted *P*) of 124 proteins using the MSstats package^29^ in R language (Supplementary Table S2). Then, volcano plots were generated for the proteins in LC versus HCC and HCC versus Recovery in the training and test sets (Supplementary Fig. S3). The overall scheme of the study is presented in Fig. 1.

The levels of 231 candidate markers were then analyzed by logistic regression (LR) in the training set (30 LC, 30 HCC, and 30 Recovery). Consequently, 38 proteins (70 peptides) in LC versus HCC were differentially expressed [area under the receiver operating curve (AUROC) values ≥0.800] (Supplementary Table S3). When the same targets were analyzed in the independent test set of 90 samples (30 LC, 30 HCC, and 30 Recovery), only 2 proteins [fibronectin (FN1) and prothrombin (THRB)] and 5 peptides had an AUROC values  ≥ 0.800 (Supplementary Table S3). The 1 peptide target was modeled in the training set and then crossvalidated in the test set for LC versus HCC and HCC versus Recovery. Among the single peptide, only FN1 had an AUROC values  ≥ 0.800 in all comparisons (Table 2). Thus, we focused on FN1, which differed significantly in LC versus HCC and HCC versus Recovery patients throughout the analyses in the training and test sets.

**Table 2:** Crossvalidation results of FN1 as measured by MRM-MS between the training and test sets. LC, liver cirrhosis; HCC, hepatocellular carcinoma; Recovery, Patients who were recovered from HCC; Spec., specificity; Sens., sensitivity.

Group		Fibronectin (Peptide Sequence)		
		GEWTCIAYSQLR	HTSVQTTSSGSGPFTDVR	WCGTTQNYDADQK
LC vs. HCC	AUROC	0.916	0.920	0.926
	Spec.	0.933	0.967	0.933
	Sens.	0.867	0.767	0.833
Recovery vs. HCC	AUROC	0.881	0.889	0.896
	Spec.	0.900	0.900	0.900
	Sens.	0.833	0.800	0.833

### Western blot analysis

**Figure 2:**
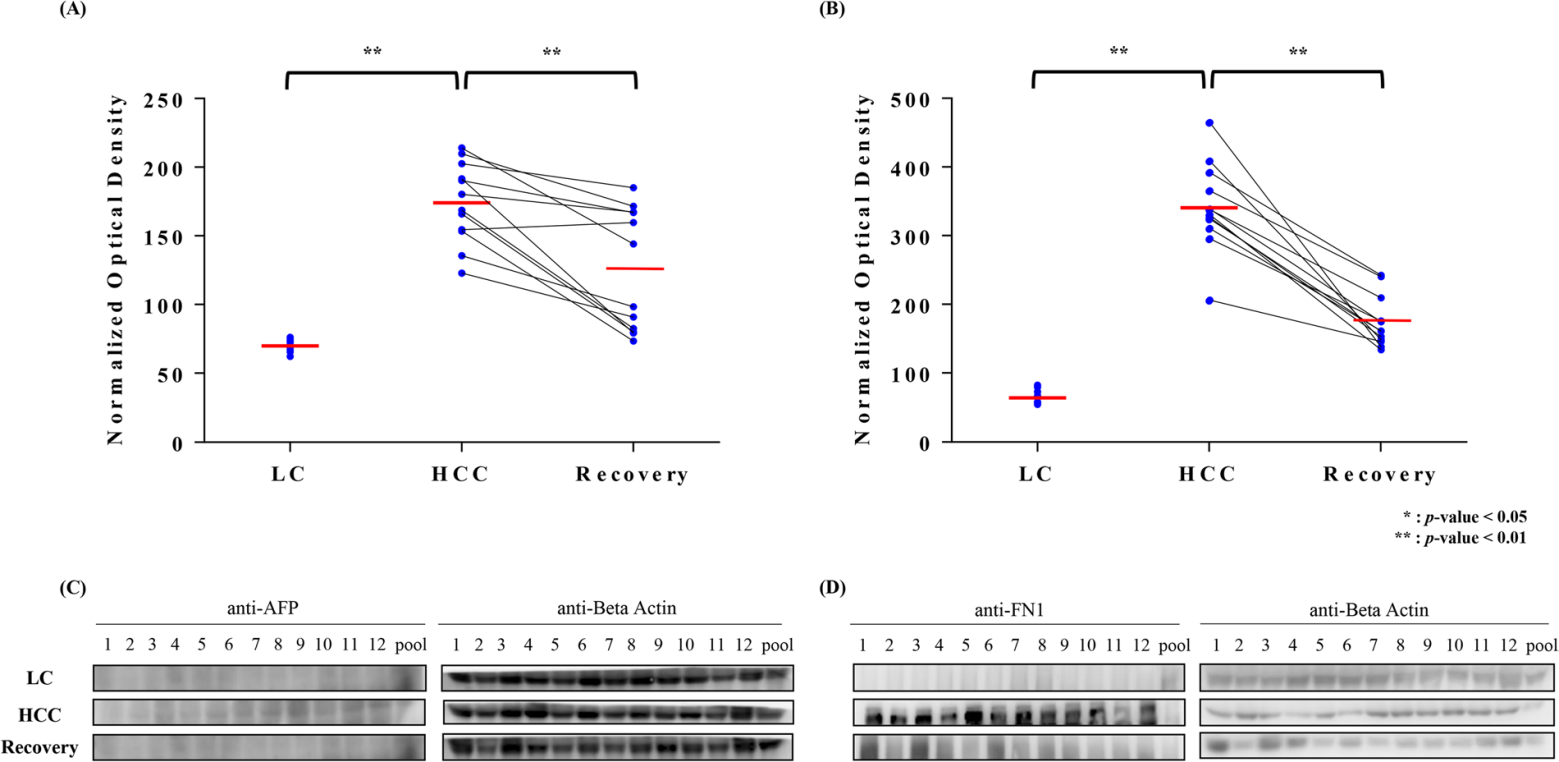
Western blot results of AFP and FN1. The difference in (**A**) AFP and (**B**) FN1 levels between the 3 groups (LC, HCC, and Recovery) is shown in the scatter plot. In addition, expression levels difference between HCC and Recovery subjects are shown in the paired plot. All individual data were normalized to that of the pooled serum sample. The middle red line is the mean. AFP and FN1 levels in HCC patient samples were higher than in LC patients. When HCC patients recovered from the cancer, these 2 proteins decreased again. Beta-actin was used as a loading control. Images of uncropped western blots can be found in Supplementary Fig. S4.

To verify the expression on the peptide level by MRM-MS analysis, the levels of selected proteins were measured by western blotting. FN1 remained high in HCC patients compared with LC and Recovery patients (Fig. 2). The normalized optical density was nearly 2 to 4 times higher in HCC than LC and Recovery patients (*P* < 0.01). Alpha-fetoprotein (AFP) expression was also elevated in HCC by approximately 2-fold versus LC (*P* < 0.01), but the decrease in AFP from HCC to Recovery (*P* < 0.01) was less extensive compared with that of FN1. The rising and downward pattern of FN1 in the LC, HCC, and Recovery groups was similar to the MRM-MS results.

### Validation of FN1 and the 2-marker panel by ELISA

**Figure 3:**
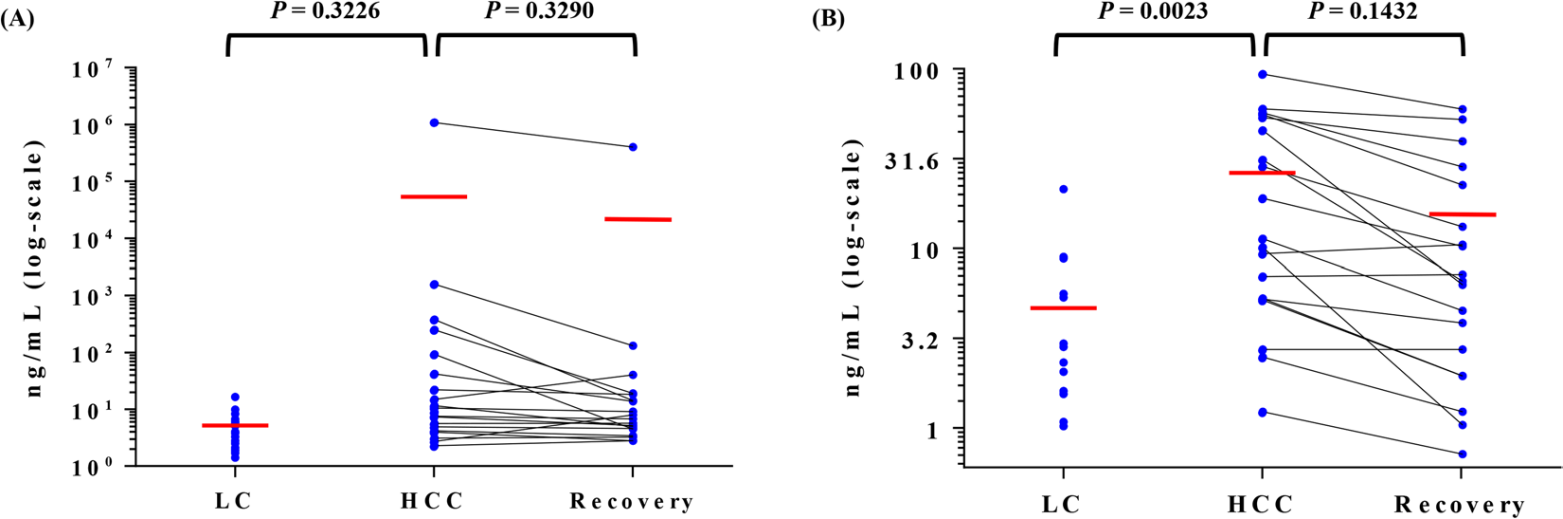
ELISA results of AFP and FN1. The difference in (**A**) AFP and (**B**) FN1 levels between the 3 groups (LC, HCC, and Recovery) is shown in the scatter plot. In addition, quantitative data difference between HCC and Recovery subjects are shown in the paired plot. The middle red line is the mean. AFP and FN1 levels in HCC patient samples were higher than in LC patients. When HCC patients recovered from the cancer, these 2 proteins decreased again. Only significant differences were observed in the LC and HCC groups compared with FN1.

**Figure 4:**
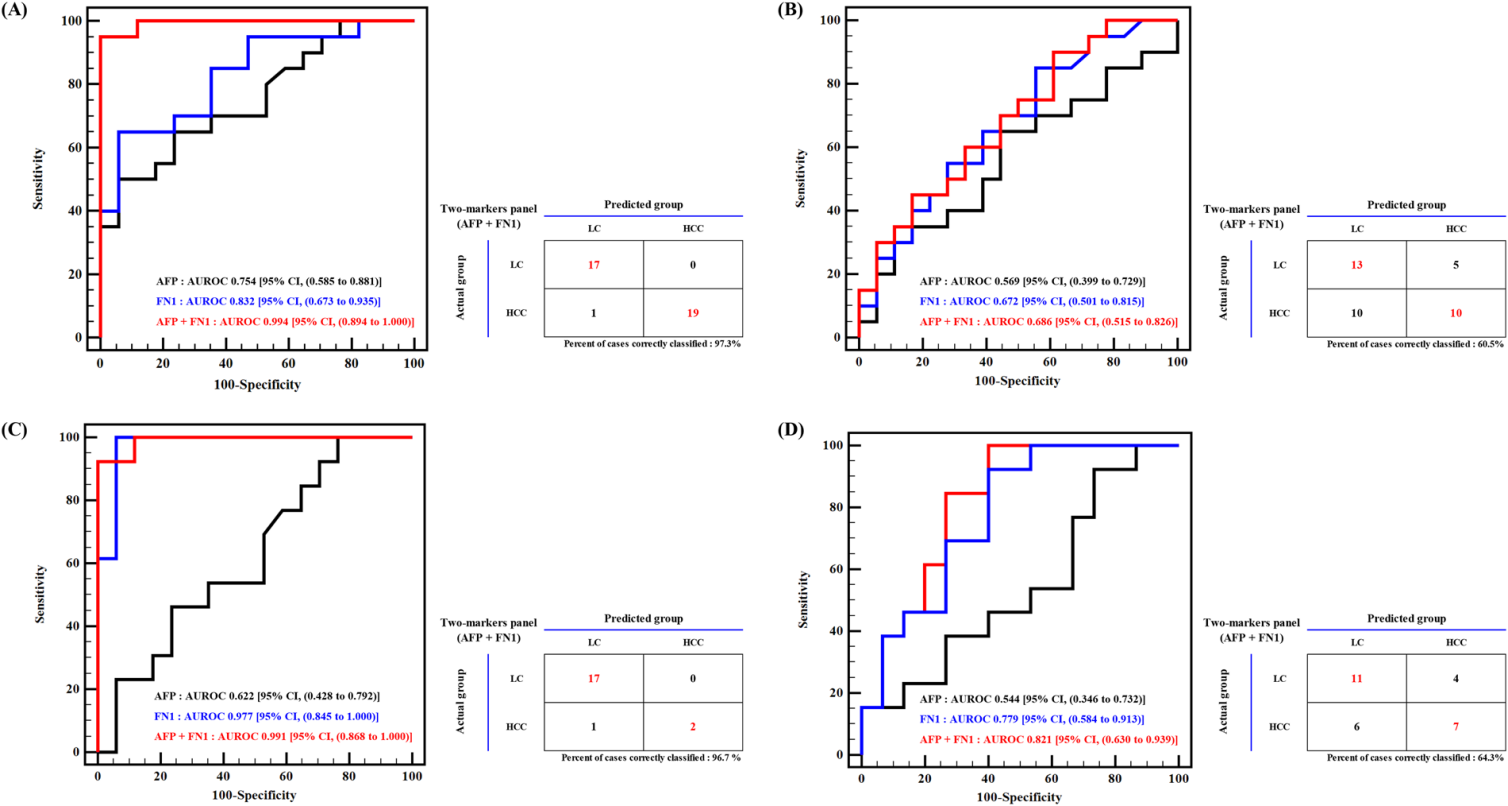
ROC curve of AFP, FN1, and AFP + FN1 in LC vs. HCC and HCC vs. Recovery by ELISA. The final target, FN1, was evaluated by ELISA in 60 independent serum samples (20 LC, 20 HCC, and 20 Recovery). The panel that was developed from AFP and FN1 enhanced the performance of the individual markers in (**A**) LC vs. HCC and (**B**) HCC vs. Recovery. AFP and FN1 distinguished HCC from LC patients with high AUROC values, but both were less effective in HCC vs. Recovery. The 2-marker panel of AFP and FN1 increased the detection rate of HCC from recovered patients. Also, the ROC curves for AFP, FN1, and AFP + FN1 were drawn only for the normal range of AFP (<20 ng/mL) patients. (**C**) LC vs. HCC and (**D**) HCC vs. Recovery. The AUROC values in each group comparison are shown at the bottom corner of the graph. As expected, AFP alone could not diagnose HCC from LC or Recovery, but FN1 improved the AUROC value by more than 0.3. Adding AFP to FN1 increased the likelihood of differentiating HCC from other benign states.

The final target, FN1, was validated by enzyme-linked immunosorbent assay (ELISA) in 60 independent serum samples (20 LC, 20 HCC, and 20 Recovery). Individual samples were measured once by ELISA. After the concentrations were measured, 3 samples from LC patients and 1 Recovery patient sample were excluded, because their results were out of the dynamic range. The levels of AFP and FN1 rose in HCC compared with LC and decreased again in the Recovery samples (Fig. 3). The ELISA results showed similar expression trends as in the MRM-MS findings. When the ELISA results for LC and HCC were analyzed by LR, the AUROC values for AFP and FN1 were 0.754 and 0.832, respectively (Fig. 4A). In addition, when the ELISA results for HCC and recovery were analyzed by LR, the AUROC values for AFP and FN1 were 0.569 and 0.672, respectively, lower than the ELISA data for LC and HCC (Fig. 4B).

The 2-marker panel of AFP and FN1 was also examined by LR. The AUROC value was 0.994 in LC versus HCC, which improved the diagnostic performance of AFP and FN1 by 0.16–0.24 in AUROC values (AUROC_AFP_ = 0.754, AUROC_FN1_ = 0.832). Approximately 97.3% of LC and HCC patients were correctly classified (Fig. 4A). In HCC versus Recovery patients, the AUROC value was 0.686, and 60.5% of cases were accurately predicted. The AUROC value of AFP alone was 0.569 (Fig. 4B). These results imply that the combination of AFP and FN1 enhances the diagnostic performance of AFP, a conventional clinical marker, and FN1, a newly discovered marker in this study.

### Performance of the 2-marker panel with normal range of AFP

The panel, comprising AFP and FN1, was tested on 20 LC, 13 HCC, and 13 Recovery serum samples to evaluate its performance in patients with normal AFP levels (<20 ng/mL). (Table 1)As expected, AFP could not differentiate LC or HCC. The ELISA results on FN1 and AFP in LC versus HCC, the AUROC values were 0.622 and 0.977, respectively (Fig. 4C). This 2-marker panel had an AUROC value of 0.991, which exceeded the performance of the individual markers. Even for HCC versus Recovery patients within the normal range of AFP, FN1 had an AUROC value of 0.779, compared with 0.544 for AFP (Fig. 4D). This 2-marker panel had an AUROC value of 0.821. Thus, we concluded that FN1 complemented AFP when these 2 markers were combined to distinguish HCC from LC and Recovery patients.

## Discussion

In this study, we developed a 2-marker panel for the early detection of hepatocellular carcinoma (HCC) and evaluation of treatment response, verifying and validating protein marker candidates using multiple reaction monitoring-mass spectrometry (MRM-MS), western blot analysis, and enzyme-linked immunosorbent assay (ELISA). The combination of alpha-fetoprotein (AFP) and fibronectin (FN1) performed better diagnostically than the current marker, AFP, alone. The serum levels of these markers increased in HCC compared with liver cirrhosis patients (LC) and declined again in the recovery of HCC patients (Recovery). These results indicate that our 2-marker panel can distinguish HCC from high-risk populations with LC and evaluate how well patients respond to the treatment, even for those with AFP levels that are within the normal range.

According to our study, the performance of FN1 exceeded that of AFP for the early detection of HCC. When FN1 was analyzed by MRM-MS, the area under the receiver operating curve (AUROC) value was 0.926 in distinguishing HCC from LC. The AUROC value remained high (AUROC = 0.832) when FN1 was measured by ELISA. Further, FN1 complements the current marker, AFP, in patients who are within the normal range of AFP. AFP alone could not diagnose HCC from LC or Recovery, but 2-marker panel improved the AUROC value by more than 0.28–0.37. This result implies that the panel that comprises AFP and FN1 performs better than the individual markers for LC versus HCC and HCC versus recovered patients.

FN1 is produced by hepatocytes and exists in the extracellular matrix^30, 31^. It binds to integrins, collagen, heparin sulfate proteoglycans, and fibrin^32–35^. As a glycoprotein, FN1 mediates cell adhesion, growth, differentiation, and migration, all of which are involved in host

defense, blood coagulation, metastasis, and wound healing^36–40^. Several studies have linked FN1 to vascular events^41, 42^. Further, lung cancer development and radioresistance in head and neck squamous cell carcinoma^43^ also involve FN1. FN1 activates mTOR signaling in gallbladder cancer^44^ and lymphedema in korean breast cancer survivors^45^. Neuropilin-1 (NRP-1) promotes α5β1 integrin-dependent FN1 fibril assembly and tumor growth by aiding myofibroblasts and soluble FN1 interactions^46^. According to the Human Protein Atlas database^47^ (http://www.proteinatlas.org/) and previous reports, staining of HCC tissues with FN1 antibody results in moderate to strong intensity, as opposed to hepatocytes and bile duct cells in normal liver, in which FN1 is low or undetected. FN1 is overexpressed in the cytoplasm or membrane of surgically resected HCC compared with adjacent normal liver tissue^48^.

FN1, which exists in plasma and cellular forms, is observed as alternatively spliced mRNA variants by immunohistochemistry and reverse transcriptase-polymerase chain reaction (RT-PCR) in liver biopsies and surgical resections. FN1 is deposited in large quantities in the extracellular space of portal areas and sinusoids when hepatocytes and nonparenchymal cells express cellular FN1 mRNA, suggesting early secretion of FN1^49^. Another report suggests that tumor cells produce FN1 and shed surface FN1, contributing to the increasing levels of FN1 in plasma or ascitic fluid. A periacinar pattern and the accumulation of FN1 around tumor nodules and the surrounding fibrous capsule have been also observed in HCC. The deposition of FN1 isotypes might be relevant to the malignant phenotype of tumor cells in favoring cell migration and proliferation^50, 51^. Thus, elevated FN1 levels in the liver might accelerate hepatic fibrogenesis and malignant alterations in HCC. The origin and function of FN1 should be examined more extensively, but our findings suggest that FN1 is highly related to HCC.

Despite the significance of our study, the major limitation was that all samples were obtained from a single institution, which might have led to institutional bias. More clinical validation is required to introduce the 2-marker panel to clinical practice. This marker should be specific for HCC patients, at least compared with other cancers. Measuring the level of FN1 in other cancers would be helpful to ensure that this target is specific solely for HCC.

On the other hand, according to Table 1 of study population, the gender distribution of LC was different from that of HCC/Recovery. The percentage of males in HCC/Recovery are much higher than in LC. To identify these issues, we exclusively used male patient data to determine whether serum fibronectin levels remained significantly different between groups (see the Supplementary Table S5). The results of the additional analyses with male patient-only data were similar to that of the whole data. Thus, we conclude that serum fibronectin concentrations in male patient-only data are still significantly different between groups and similar to that of the whole data.

Our study indicates that the early stages [tumor, node, and metastasis (TNM) stage I and II] of HCC can be diagnosed using the combination of AFP and FN1 through a routine blood test. This 2-marker panel can guide the appropriate medical decisions regarding imaging or treatment. Further, a postsurgical examination should be possible as the level of FN1 declines, when patients recover from HCC. Examining the pathways in which FN1 participates and the mechanisms by which FN1 functions in HCC might help identify potential targets for its treatment and increase our understanding of the development of HCC.

## Methods

### Materials

Trypsin was obtained from Promega (sequencing-grade modified, WI, USA). High-performance liquid chromatography (HPLC)-grade water and acetonitrile were purchased from Thermo Fisher Scientific (Bremen, Germany). Serum depletion was performed for the 6 most abundant proteins using a multiple affinity removal system (MARS), consisting of an LC column (Agilent, CA, USA); buffer A (Agilent, CA, USA) for sample loading, washes, and equilibration; and buffer B (Agilent, CA, USA) for elution. Unpurified stable isotope-labeled standard (SIS) peptides [isotopically labeled (^13^C and^15^N) amino acids] were obtained from JPT (Berlin, Germany) (30% to 70% purity, according to the manufacturer).

### Study participants

A total of 240 serum samples from liver cirrhosis (LC) and hepatocellular carcinoma (HCC) patients and patients who recovered from HCC (Recovery) were collected from 2008 to 2014 (Table 1). The serum was separated using a serum-separating tube. HCC was staged based on the tumor, node, and metastasis (TNM) staging system, which classifies a solitary tumor without vascular invasion as stage I and multiple tumors that are <5 cm or a solitary tumor with vascular invasion as stage II. Recovery from HCC was defined as complete remission of the tumor after treatment without recurrence of the cancer for 6 months. All recovered patients were examined by computed tomography or magnetic resonance imaging. All samples were collected

from Seoul National University Hospital (Seoul, Republic of Korea), per the same standard operating procedures^52^. The samples were stored at −70 °C until processing.

This study was approved by the institutional review board (IRB) of Seoul National University Hospital (IRB approval No. 0506-150-005), and all patients provided informed consent before being enrolled in this study. All experiments were performed in accordance with relevant guidelines and regulations.

### Clinical sample preparation for MRM-MS analysis

All clinical samples were depleted of high-abundance proteins on a Multiple Affinity Removal System Human-6 (MARS Hu-6, 4.6 mm × 100 mm, Agilent, CA, USA) affinity column that was connected to an HPLC instrument (Shimadzu, Kyoto, Japan). Depleted serum sample was concentrated, and 0.1 mg of depleted serum was denatured with 6 M urea. After the trypsin digestion and desalting steps, the eluted sample was lyophilized on a vacuum centrifuge. All samples were stored at −80 °C and resolubilized in 0.1% formic acid/water until the multiple reaction monitoring-mass spectrometry (MRM-MS) analysis (more details are given in Supplementary Methods of Supplementary Information).

### Quantitative MRM-MS analysis

All MRM-MS assays were performed on an Agilent 6490 triple quadrupole (QQQ) mass spectrometer (Agilent, CA, USA) with a Jetstream electrospray source that was equipped with a 1260 Infinity HPLC system (Agilent, CA, USA). The reversed-phase analytical column (150 mm × 0.5 mm id, Agilent Zorbax SB-C18, 3.5-µm particle size) was maintained at 40 °C, and a binary gradient of 3% to 35% acetonitrile/0.1% formic acid flowed through the column for 45 minutes at 10 µL/min to separate the peptides. Mobile phases A and B were composed of 0.1% formic acid/water (v/v) and 0.1% formic acid/acetonitrile (v/v), respectively. The MRM-MS assay was conducted in positive mode. The ion spray capillary voltage was set to 2500 V, and the nozzle voltage was 2000 V. The cell accelerator voltage was adjusted to 5 V, the delta electron multiplier voltage (EMV) was 200 V, and the fragment voltage was 380 V. The temperature of the drying gas was 250 °C at 15 L/min, and the sheath gas was set to 350 °C at a flow rate of 12 L/min. All MRM-MS raw data files were processed in Skyline (McCoss Lab, University of Washington, USA).

### Western blot analysis

To verify the differential expression of proteins at the peptide level by MRM-MS, the concentrations of selected proteins were further examined by western blot. We analyzed 12 LC, 12 HCC, 12 Recovery samples, and 1 pooled sample from all 36 patients by western blot to confirm the MRM-MS data. All samples were selected from the test set (n = 90). Alpha-fetoprotein (AFP) and fibronectin (FN1) were examined by sodium dodecyl sulfate-polyacrylamide gel electrophoresis (SDS-PAGE). Pre-stained protein marker was loaded into the first lane, followed by 12 individual serum samples and the pooled serum sample. We included beta-actin as control for loading amounts. The proteins were transferred to a nitrocellulose membrane at 100 V for 1 hr. Buffer solution was added to the apparatus to maintain the appropriate temperature. Nonspecific binding was blocked with bovine serum albumin (BSA), and the primary antibody was added overnight. The primary antibodies targeted AFP (1:100; Santa Cruz, CA, USA), fibronectin (1:50,000; Abcam, MA, USA), and beta-actin (1:10,000; Abcam, MA, USA). After the membrane was washed, the signals were visualized on an LAS-4000 mini Luminescent image analyzer (Fujifilm, Tokyo, Japan). The intensities of all samples were normalized to that of the pooled serum sample to adjust for the variation in between gels. The results were similar to those of MRM-MS (Fig. 2).

### Validation by ELISA

The serum level of FN1 was validated in independent samples of 20 LC, 20 HCC, and 20 Recovery patients (“Validation set”) using ELISA kit (USCN, TX, USA). The serum was diluted 10,000-fold, and 100 µL of the serum sample was loaded into each well. After being incubated for 2 hours at 37 °C, the wells were washed and incubated with Detection Reagent A. After 1 hour of incubation at 37 °C and a wash step, Detection Reagent B was added for 30 min, after which the wells were washed and incubated with substrate solution. A stop solution ceased the reaction after 20 min, and the density was measured at 450 nm. The concentration of FN1 was calculated by drawing a 7-point calibration curve and multiplying by the dilution factor.

### Statistical analysis

The raw MRM-MS data were processed in Skyline to calculate the peak areas of the transitions. The relative abundance of the best transition was determined, based on the normalized peak areas of the endogenous peptide to the unpurified SIS peptide. All transition intensities were base 2 log-transformed to construct the prediction model. The data points were smoothed by Savitzky-Golay method^53^. The performance of the biomarkers was evaluated using area under the receiver operating curve (AUROC), sensitivity, and specificity^54^. Single- and multimarker analyses by MRM-MS and ELISA were performed by logistic regression^55^ to determine the best predictive model. Three-fold crossvalidation was repeated 100 times in the training and test sets. The MRM-MS, western blot, and ELISA results were analyzed by t-test (independent or paired), and *P* < 0.05 were considered to be significant. Relative abundance of the proteins were calculated using the linear mixed effects model (LMM) in MSstats. All statistical analyses were performed using R language, ver. 3.2.1 (R Foundation for Statistical Computing, Vienna, Austria), IBM SPSS, ver. 19.0 (SPSS Inc., IL, USA), and Graph Pad Prism, ver. 6.0 (Graph PAD, CA, USA).

### Data availability

All data generated during this study are included in this published article and its Supplementary Information file.

## Electronic supplementary material

Supplementary Information
