# Supplementary material for: Serum fibronectin distinguishes the early stages of hepatocellular carcinoma
Source: Sci Rep. 2017 Aug 25;7:9449. doi: 10.1038/s41598-017-09691-3 (PMC5573357; doi:10.1038/s41598-017-09691-3)

Serum fibronectin distinguishes the early stages of hepatocellular carcinoma

Hyunsoo Kim[1,2,3,‡], JiYoung Park[1,‡], Yongkang Kim[4,‡], Areum Sohn[1], Injun Yeo[2], Su Jong Yu[5], Jung-Hwan Yoon[5], Taesung Park[4,], and Youngsoo Kim[1,2,3,*]*

SUPPLEMENTARY INFORMATION

Supplementary Methods

Experimental design

To verify the protein markers that complemented the early diagnosis of HCC, we quantitated candidate markers in the serum of patients with liver cirrhosis, HCC, and recovery from HCC. All serum samples were collected retrospectively from the same institution. A list of previously discovered proteins from the LiverAtlas database was measured by multiple reaction monitoring-mass spectrometry (MRM-MS), a linear mixed effects model (LMM), and logistic regression (LR) methods were used to select significant markers of HCC. The performance of the targets was based on their area under the receiver operating curve (AUROC). Differentially expressed proteins were also verified by western blot. The final target, fibronectin (FN1), was validated by enzyme-linked immunosorbent assay (ELISA) to ensure its differential expression. The number of samples was based on an AUROC values ≥ 0.800 with 95% confidence interval (CI), because the AUROC value of AFP has been reported to be 0.50 – 0.76[1-3].

The α (significance) and β (1-power) were 0.05. The ratio of cases to controls was set to 1:1, and a total of 40 samples, composed of 20 cases and 20 controls, were needed (Supplementary Table S4). In this study, 240 samples, comprising 80 cases and 160 controls, were collected. We negated the subjectivity of experimenters by blocked randomization. Equal numbers of control and case samples were assigned in each randomly ordered block using our best effort using Excel, ver. 2013 (Microsoft Corp., WA, USA). Random allocation was processed using Random Allocation Software, ver. 1.00 (Isfahan University of Medical Sciences,

Isfahan, Iran). We block-randomized the 3 steps (depletion order, digestion order, order of analysis by MRM-MS) to prevent experimental bias.

Clinical sample preparation for MRM-MS analysis

The high-abundance proteins albumin, IgG, IgA, haptoglobin, transferrin, and alpha-1-antitrypsin were depleted using a Multiple Affinity Removal System Human-6 (MARS Hu-6, 4.6 mm × 100 mm, Agilent, CA, USA) affinity column on a high-performance liquid chromatography (HPLC) instrument to remove any masking effects and discover low-abundance targets. Serum sample were thawed on ice and centrifuged at 14,000 *g* for 10 min at 4°C. For each sample, 40 µL of each serum was diluted with 160 µL MARS buffer A (Agilent, CA, USA) and passed through 0.22 µm Spin-X filters (Corning Costar, NY, USA). The diluted serum protein was then processed using the recommended column run cycle. The specific process was as follows: After equilibration with buffer A (load/wash buffer), the MARS column was loaded with 200 µL of the diluted serum at a flow rate of 0.5 mL/min for 10 min. Flow-through fractions, representing depleted serum, were eluted at 3 min. The bound proteins were released with 100% buffer B (elution buffer) at 16.5 min with a flow rate of 1.0 mL/min. Each depletion cycle took 28 min of total run time.

The depleted serum was then concentrated on 3000 Da molecular weight cutoff (MWCO) centrifugal filter units (Amicon Ultra-4 3K, Millipore, MA, USA). One hundred micrograms of concentrated serum, as measured by bicinchoninic acid assay, was denatured with 6 M urea. The reduction and alkylation were performed with 20 mM dithiothreitol (Merk, Darmstadt, Germany), 0.1 M Tris, pH 8.0 at 37°C for 30 min and 50 mM iodoacetamide (Sigma, MO, USA) in the dark at room temperature for 30 min, respectively. To enhance the digestion with sequencing-grade

trypsin (Promega, WI, USA) at a 1:50 enzyme-to-substrate ratio, the serum was diluted 10-fold with 0.1 M Tris, pH 8.0 and incubated for 16 h at 37°C. To cease the enzymatic reaction, neat formic acid was added until a final concentration of 2%.

In the desalting step, Oasis® HLB 1 cc (30 mg) extraction cartridges (Waters Corp., MA, USA) were washed with 1 mL 100% methanol and 3 mL 100% acetonitrile in 0.1% formic acid. The digested sample was loaded onto the cartridge after equilibration with 3 mL 0.1% formic acid. The cartridge was then washed with 3 mL 0.1% formic acid and eluted with 1 mL 80% acetonitrile in 0.1% formic acid. The collected sample was lyophilized on a vacuum centrifuge. The serum was stored at -80°C and resolubilized in 0.1% formic acid to 2 µg/µL prior to the MRM-MS analysis.

References

1. Dong, X. *et al.* Combination of serum RASSF1A methylation and AFP is a promising non-invasive biomarker for HCC patient with chronic HBV infection. *Diagnostic pathology* **10**, 133, doi:10.1186/s13000-015-0317-x (2015).
2. Wan, H. G. *et al.* Comparison osteopontin vs AFP for the diagnosis of HCC: a meta-analysis. *Clin Res Hepatol Gastroenterol* **38**, 706-714, doi:10.1016/j.clinre.2014.06.008 (2014).
3. Ji, J. *et al.* Diagnostic Evaluation of Des-Gamma-Carboxy Prothrombin versus alpha-Fetoprotein for Hepatitis B Virus-Related Hepatocellular Carcinoma in China: A Large-Scale, Multicentre Study. *PloS one* **11**, e0153227, doi:10.1371/journal.pone.0153227 (2016).

Supplementary Figure Legends

Supplementary Figure S1. MRM-MS results in training set samples of AFP and FN1.

The difference in quantitative data between the 3 groups (LC, HCC, and Recovery) for AFP peptides (A) AENAVECFQTK, and (B) GYQELLEK are shown along with that of FN1 peptides (C) GEWTCIAYSQLR, (D) HTSVQTTSSGSGPFTDVR, and (E) WCGTTQNYDADQK in the scatter plot. In addition, quantitative data difference between HCC and Recovery subjects are shown in the paired plot. All individual data were normalized (peak area of the endogenous peptide/peak area of the unpurified SIS peptide) and then averaged over 3 repeat measurements. The middle red line is the mean.

Supplementary Figure S2. MRM-MS results in test set samples of AFP and FN1.

The difference in quantitative data between the 3 groups (LC, HCC, and Recovery) for AFP peptides (A) AENAVECFQTK, and (B) GYQELLEK are shown along with that of FN1 peptides (C) GEWTCIAYSQLR, (D) HTSVQTTSSGSGPFTDVR, and (E) WCGTTQNYDADQK in the scatter plot. In addition, quantitative data difference between HCC and Recovery subjects are shown in the paired plot. All individual data were normalized (peak area of the endogenous peptide/peak area of the unpurified SIS peptide) over 1 measurement. The middle red line is the mean.

Supplementary Figure S3. Volcano plot of 124 proteins in LC vs. HCC and HCC vs. Recovery. Statistical significance against fold-change (x-axis) and adjusted *P* (y-axis) between (A,

C) LC versus HCC patients and (B, D) HCC versus Recovery patients (A, B) in the training and (C, D) test sets, respectively, in which FN1 is significantly differentially expressed.

Supplementary Figure S4. Uncropped western blots. (A) AFP and (B) FN1 were western blotted in LC group (upper blots), HCC group (middle blots), and Recovery group (lower blots). Left blots were for target proteins; AFP and FN1, and right blots for control protein (beta-actin). The red boxes indicate the cropped regions.

Supplementary Table S1. Automated detection of inaccurate and imprecise transitions (AuDIT) analysis for 231 peptides (124 proteins) and measurements of endogenous peptide concentrations.

AuDIT, automated detection of inaccurate and imprecise transitions; STDEV, standard deviation; m/z, mass-to-charge ratio; CV, coefficient of variation.

Number	Target information					Mass information					Result of AuDIT		Endogenous level
	Gene Symbol	Uniprot Accession Number	Uniprot ID	Protein Name	Peptide Sequence	Precursor ion (m/z)	Precursor ion charge	Product ion (m/z)	Product ion charge	Product ion type	*P*	CV	fmol
1	SFN	P31947	1433S	14-3-3 protein sigma	NLLSVAYK	454.3	2	680.4	1	y6	0.816	0.043	5.866
2	SERPIN A1	P01009	A1AT	Alpha-1-antitrypsin	SVLGQLGITK	508.3	2	829.5	1	y8	0.982	0.041	91.146
3	SERPIN F2	P08697	A2AP	Alpha-2-antiplasmin	LCQDLGPGAFR	617.3	2	604.3	1	y6	0.109	0.021	177.811
4	SERPIN F2	P08697	A2AP	Alpha-2-antiplasmin	WFLLEQPEIQVAHFPFK	710.4	3	334.2	1	b2	0.820	0.019	579.287
5	LRG1	P02750	A2GL	Leucine-rich alpha-2-glycoprotein	ENQLEVLEVSWLHGLK	632.0	3	705.4	2	y12	0.842	0.063	1267.102
6	LRG1	P02750	A2GL	Leucine-rich alpha-2-glycoprotein	DLLLPQPDLR	590.3	2	725.4	1	y6	0.765	0.070	266.010
7	LRG1	P02750	A2GL	Leucine-rich alpha-2-glycoprotein	VAAGAFQGLR	495.3	2	819.4	1	y8	0.947	0.039	159.117
8	A2M	P01023	A2MG	Alpha-2-macroglobulin	NEDSLVFVQTDK	697.8	2	737.4	1	y6	0.701	0.067	16752.126
9	A2M	P01023	A2MG	Alpha-2-macroglobulin	HNVYINGITYTPVSSTNEK	1069.0	2	861.4	1	y8	0.707	0.028	21134.691
10	A2M	P01023	A2MG	Alpha-2-macroglobulin	TEHPFTVEEFVLPK	558.3	3	357.2	1	y3	0.105	0.074	55709.830
11	A2M	P01023	A2MG	Alpha-2-macroglobulin	NQGNTWLTAFVLK	497.9	3	351.2	2	b6	0.064	0.097	22971.806
12	SERPIN A3	P01011	AACT	Alpha-1-antichymotrypsin	DEELSCTVVELK	711.3	2	374.1	1	b3	0.475	0.060	442.211
13	SERPIN A3	P01011	AACT	Alpha-1-antichymotrypsin	ADLSGITGAR	480.8	2	574.3	1	y6	0.101	0.037	2919.600
14	IGFALS	P35858	ALS	Insulin-like growth factor-binding protein complex acid labile subunit	NLIAAVAPGAFLGLK	727.9	2	802.5	1	y8	0.391	0.046	65.722
15	IGFALS	P35858	ALS	Insulin-like growth factor-binding protein complex acid labile subunit	LEYLLLSR	503.8	2	764.5	1	y6	0.945	0.026	47.287
16	IGFALS	P35858	ALS	Insulin-like growth factor-binding protein complex acid labile subunit	ANVFVQLPR	522.3	2	759.5	1	y6	0.106	0.068	54.274
17	IGFALS	P35858	ALS	Insulin-like growth factor-binding protein complex acid labile subunit	LEALPNSLLAPLGR	732.4	2	1037.6	1	y10	0.586	0.027	64.787
18	AMBP	P02760	AMBP	Protein AMBP	TVAACNLPIVR	607.3	2	484.3	1	y4	0.644	0.059	1958.766
19	AMBP	P02760	AMBP	Protein AMBP	AFIQLWAFDAVK	704.9	2	836.4	1	y7	0.628	0.119	14391.065
20	AGT	P01019	ANGT	Angiotensinogen	DPTFIPAPIQAK	649.4	2	724.4	1	y7	0.528	0.061	184.132

21	AGT	P01019	ANGT	Angiotensinogen	LQAILGVPWK	562.8	2	883.5	1	y8	0.621	0.083	5.539
22	SERPIN C1	P01008	ANT3	Antithrombin-III	TSDQIHFFFAK	670.8	2	796.4	1	y6	0.963	0.069	867.968
23	SERPIN C1	P01008	ANT3	Antithrombin-III	IEDGFSLK	454.7	2	666.3	1	y6	0.907	0.055	77.930
24	APOA1	P02647	APOA1	Apolipoprotein A-I	DLATVYVDVLK	618.3	2	736.4	1	y6	0.164	0.065	12237.650
25	APOA1	P02647	APOA1	Apolipoprotein A-I	DYVSQFEGSALGK	467.6	3	532.3	1	y6	0.117	0.025	14908.533
26	APOA1	P02647	APOA1	Apolipoprotein A-I	LLDNWDSVTSTFSK	806.9	2	971.5	1	y9	0.624	0.035	50170.550
27	APOA1	P02647	APOA1	Apolipoprotein A-I	EQLGPVTQEFWDNLEK	967.0	2	753.4	2	y12	0.217	0.043	3743.703
28	APOA1	P02647	APOA1	Apolipoprotein A-I	AELQEGAR	437.2	2	673.4	1	y6	0.661	0.038	10731.523
29	APOA1	P02647	APOA1	Apolipoprotein A-I	THLAPYSDELR	434.6	3	619.3	1	y5	0.925	0.018	39828.748
30	APOA1	P02647	APOA1	Apolipoprotein A-I	ATEHLSTLSEK	405.9	3	572.8	2	y10	0.296	0.033	50718.057
31	APOA4	P06727	APOA4	Apolipoprotein A-IV	IDQNVEELK	544.3	2	974.5	1	y8	0.969	0.151	113.337
32	APOA4	P06727	APOA4	Apolipoprotein A-IV	ISASAEELR	488.3	2	775.4	1	y7	0.388	0.109	495.206
33	APOC1	P02654	APOC1	Apolipoprotein C-I	EFGNTLEDK	526.7	2	391.2	1	y3	0.133	0.084	1245.502
34	APOC1	P02654	APOC1	Apolipoprotein C-I	EWFSETFQK	601.3	2	739.4	1	y6	0.903	0.076	774.932
35	APOC2	P02655	APOC2	Apolipoprotein C-II	ESLSSYWESAK	643.8	2	870.4	1	y7	0.178	0.068	1009.418
36	APOC2	P02655	APOC2	Apolipoprotein C-II	TYLPAVDEK	518.3	2	658.3	1	y6	0.124	0.018	1178.879
37	APOC3	P02656	APOC3	Apolipoprotein C-III	DALSSVQESQVAQQAR	858.9	2	573.3	1	y5	0.111	0.036	8308.591
38	APOC3	P02656	APOC3	Apolipoprotein C-III	GWVTDGFSSLK	598.8	2	854.4	1	y8	0.998	0.024	1902.679
39	APOC4	P55056	APOC4	Apolipoprotein C-IV	ELLETVVNR	536.8	2	588.3	1	y5	0.642	0.112	10.409
40	APOC4	P55056	APOC4	Apolipoprotein C-IV	AWFLESK	440.7	2	623.3	1	y5	0.120	0.093	46.090
41	APOE	P02649	APOE	Apolipoprotein E	WELALGR	422.7	2	529.3	1	y5	0.994	0.072	407.772
42	APOE	P02649	APOE	Apolipoprotein E	AATVGSLAGQPLQER	749.4	2	827.4	1	y7	0.347	0.063	593.983
43	APOE	P02649	APOE	Apolipoprotein E	LGPLVEQGR	484.8	2	399.7	2	y7	0.979	0.055	668.426
44	APOE	P02649	APOE	Apolipoprotein E	LQAEAFQAR	517.3	2	792.4	1	y7	0.888	0.099	644.583
45	APOF	Q13790	APOF	Apolipoprotein F	SGVQQLIQYYQDQK	849.4	2	972.4	1	y7	0.100	0.031	806.278
46	APOH	P02749	APOH	Beta-2-glycoprotein 1	VCPFAGILENGAVR	751.9	2	622.3	2	y12	0.156	0.014	5647.544
47	APOH	P02749	APOH	Beta-2-glycoprotein 1	EHSSLAFWK	552.8	2	838.4	1	y7	0.995	0.152	1949.458
48	APOL1	O14791	APOL1	Apolipoprotein L1	LNILNNNYK	553.3	2	765.4	1	y6	0.627	0.031	136.007
49	APOM	O95445	APOM	Apolipoprotein M	SLTSCLDSK	505.7	2	810.4	1	y7	0.066	0.068	460.040
50	APOM	O95445	APOM	Apolipoprotein M	AFLLTPR	409.3	2	486.3	1	y4	0.684	0.009	349.394
51	TGFBI	Q15582	BGH3	Transforming growth factor-beta-induced protein ig-h3	LTLLAPLNSVFK	658.4	2	804.5	1	y7	0.167	0.020	15.425
52	BTD	P43251	BTD	Biotinidase	ILSGDPYCEK	591.3	2	955.4	1	y8	0.484	0.070	29.444
53	BTD	P43251	BTD	Biotinidase	LSSGLVTAALYGR	654.4	2	751.4	1	y7	0.922	0.048	57.844
54	CD163	Q86VB7	C163A	Scavenger receptor cysteine-rich type 1 protein M130	LVDGVTECSGR	596.8	2	980.4	1	y9	0.066	0.046	6.445
55	C1QB	P02746	C1QB	Complement C1q subcomponent subunit B	LEQGENVFLQATDK	531.3	3	675.4	1	y6	0.740	0.056	418.114

56	C1QB	P02746	C1QB	Complement C1q subcomponent subunit B	IAFSATR	383.2	2	581.3	1	y5	0.215	0.066	300.796
57	C1QC	P02747	C1QC	Complement C1q subcomponent subunit C	FQSVFTVTR	542.8	2	809.5	1	y7	0.931	0.107	603.800
58	C1QC	P02747	C1QC	Complement C1q subcomponent subunit C	TNQVNSGGVLLR	629.3	2	815.5	1	y8	0.500	0.035	284.731
59	C1RL	Q9NZP8	C1RL	Complement C1r subcomponent-like protein	GSEAINAPGDNPAK	670.8	2	698.3	1	y7	0.103	0.015	35.100
60	C1RL	Q9NZP8	C1RL	Complement C1r subcomponent-like protein	WILTAAHTIYPK	707.4	2	1114.6	1	y10	0.854	0.033	89.689
61	C4BPA	P04003	C4BPA	C4b-binding protein alpha chain	TWYPEVPK	510.3	2	732.4	1	y6	0.585	0.068	186.039
62	C4BPA	P04003	C4BPA	C4b-binding protein alpha chain	YTCLPGYVR	564.8	2	591.3	1	y5	0.100	0.026	423.746
63	C4BPA	P04003	C4BPA	C4b-binding protein alpha chain	LSLEIEQLELQR	735.9	2	915.5	1	y7	0.322	0.041	1875.149
64	C4BPB	P20851	C4BPB	C4b-binding protein beta chain	EVEGQILGTYVCIK	804.9	2	840.4	1	y7	0.334	0.103	122.620
65	C4BPB	P20851	C4BPB	C4b-binding protein beta chain	ALLAFQESK	503.8	2	709.4	1	y6	0.814	0.025	41.826
66	CTSB	P07858	CATB	Cathepsin B	EQWPQCPTIK	643.8	2	843.4	1	y7	0.272	0.082	6.172
67	CPB2	Q96IY4	CBPB2	Carboxypeptidase B2	YPLYVLK	448.3	2	366.7	2	y6	0.295	0.028	45.355
68	CPB2	Q96IY4	CBPB2	Carboxypeptidase B2	DTGTYGFLLPER	684.8	2	401.2	1	y3	0.104	0.051	24.501
69	CD5L	O43866	CD5L	CD5 antigen-like	NTCNHDEDTWVECEDPFDL R	851.3	3	647.4	1	y5	0.583	0.091	446.360
70	CD5L	O43866	CD5L	CD5 antigen-like	DVAVLCR	416.7	2	618.3	1	y5	0.983	0.095	3.666
71	CD5L	O43866	CD5L	CD5 antigen-like	LVGGDNLCSGR	574.3	2	935.4	1	y9	0.625	0.037	59.321
72	CETP	P11597	CETP	Cholesteryl ester transfer protein	ASYPDITGEK	540.8	2	759.4	1	y7	0.919	0.105	9.811
73	CFH	P08603	CFAH	Complement factor H	EGWIHTVCINGR	481.2	3	619.3	1	y5	0.391	0.062	709.656
74	CFI	P05156	CFAI	Complement factor I	VFSLQWGEVK	596.8	2	946.5	1	y8	0.718	0.064	276.430
75	CHI3L1	P36222	CH3L1	Chitinase-3-like protein 1	ILGQQVPYATK	609.3	2	991.5	1	y9	0.308	0.080	3.253
76	CHI3L1	P36222	CH3L1	Chitinase-3-like protein 1	FPLTNAIK	452.3	2	659.4	1	y6	0.345	0.112	2.620
77	BCHE	P06276	CHLE	Cholinesterase	IFFPGVSEFGK	614.3	2	967.5	1	y9	0.255	0.017	15.915
78	BCHE	P06276	CHLE	Cholinesterase	YLTLNTESTR	599.3	2	921.5	1	y8	0.951	0.012	24.973
79	CLU	P10909	CLUS	Clusterin	EIQNAVNGVK	536.3	2	415.2	2	y8	0.483	0.075	1042.401
80	CLU	P10909	CLUS	Clusterin	ASSIIDELFQDR	697.4	2	922.4	1	y7	0.100	0.015	924.827
81	CNDP1	Q96KN2	CNDP1	Beta-Ala-His dipeptidase	ALEQDLPVNIK	620.4	2	570.4	1	y5	0.329	0.063	29.102
82	CNDP1	Q96KN2	CNDP1	Beta-Ala-His dipeptidase	AIHLDLEEYR	420.2	3	467.2	1	y3	0.071	0.057	19.020
83	C2	P06681	CO2	Complement C2	GALISDQWVLTAAHCFR	649.0	3	795.9	2	y13	0.224	0.007	302.355
84	C4A	P0C0L4	CO4A	Complement C4-A	LQETSNWLLSQQQADGSFQ DPCPVLDR	1044.8	3	856.4	1	y7	0.865	0.106	780.112
85	C4A	P0C0L4	CO4A	Complement C4-A	DSSTWLTAFVLK	684.4	2	791.5	1	y7	0.100	0.015	2093.704
86	C4A	P0C0L4	CO4A	Complement C4-A	VLSLAQEQVGGSPEK	771.4	2	373.2	1	y3	0.051	0.043	1526.717
87	C4B	P0C0L5	CO4B	Complement C4-B	LQETSNWLLSQQQADGSFQ DLSPVIHR	1033.2	3	621.4	1	y5	0.495	0.188	3607.859

88	C5	P01031	CO5	Complement C5	NADYSYSVWK	616.8	2	932.5	1	y7	0.767	0.100	620.049
89	C5	P01031	CO5	Complement C5	GGSASTWLTAFALR	719.4	2	791.5	1	y7	0.929	0.029	188.797
90	C6	P13671	CO6	Complement component C6	ALNHLPLEYNSALYSR	621.0	3	425.2	1	y3	0.164	0.039	166.314
91	C6	P13671	CO6	Complement component C6	GGNQLYCVK	519.8	2	569.3	1	y4	0.506	0.069	158.231
92	C7	P10643	CO7	Complement component C7	LSGNVLSYTFQVK	728.4	2	985.5	1	y8	0.688	0.031	265.937
93	C7	P10643	CO7	Complement component C7	DSCTLPASAEK	589.8	2	602.3	1	y6	0.437	0.023	340.825
94	C7	P10643	CO7	Complement component C7	VLFYVDSEK	550.3	2	887.4	1	y7	0.818	0.085	614.780
95	C8B	P07358	CO8B	Complement component C8 beta chain	YEFILK	406.7	2	520.3	1	y4	0.630	0.033	52.176
96	C8B	P07358	CO8B	Complement component C8 beta chain	IPGIFELGISSQSDR	809.9	2	753.4	2	y14	0.987	0.100	216.211
97	C8B	P07358	CO8B	Complement component C8 beta chain	SGFSFGFK	438.7	2	585.3	1	y5	0.538	0.044	56.002
98	COLEC11	Q9BWP8	COL11	Collectin-11	VFIGINDLEK	574.3	2	901.5	1	y8	0.469	0.127	4.894
99	CPN2	P22792	CPN2	Carboxypeptidase N subunit 2	TLNLAQNLLAQLPEELFHPLTSLQTLK	1015.9	3	1089.6	2	y19	0.297	0.028	369.097
100	CPN2	P22792	CPN2	Carboxypeptidase N subunit 2	LSNNALSGLPQGVFGK	534.6	3	366.7	2	y7	0.093	0.026	206.726
101	CRTAC1	Q9NQ79	CRAC1	Cartilage acidic protein 1	SSPYYALR	478.7	2	391.7	2	y6	0.790	0.131	9.115
102	CRP	P02741	CRP	C-reactive protein	ESDTSYVSLK	564.8	2	347.2	1	y3	0.999	0.024	188.949
103	PPBP	P02775	CXCL7	Platelet basic protein	NIQSLEVIGK	550.8	2	873.5	1	y8	0.106	0.090	933.611
104	PPBP	P02775	CXCL7	Platelet basic protein	GTHCNQVEVIATLK	523.9	3	773.5	1	y7	0.986	0.057	1934.298
105	F10	P00742	FA10	Coagulation factor X	ETYDFDIAVLR	671.3	2	686.4	1	y6	0.781	0.114	67.288
106	F11	P03951	FA11	Coagulation factor XI	DSVTETLPR	509.3	2	716.4	1	y6	0.459	0.038	12.324
107	F11	P03951	FA11	Coagulation factor XI	ALSGFSLQSCR	613.3	2	1041.5	1	y9	0.144	0.055	18.117
108	F12	P00748	FA12	Coagulation factor XII	NWGLGGHAFCR	425.5	3	487.7	2	y9	0.960	0.124	92.970
109	F12	P00748	FA12	Coagulation factor XII	VVGGLVALR	442.3	2	685.4	1	y7	0.965	0.143	87.485
110	F9	P00740	FA9	Coagulation factor IX	NCELDVTCNIK	683.3	2	849.4	1	y7	0.432	0.039	96.515
111	F9	P00740	FA9	Coagulation factor IX	SALVLQYLR	531.8	2	692.4	1	y5	0.859	0.134	17.044
112	FBLN1	P23142	FBLN1	Fibulin-1	DCSLPYATESK	635.8	2	795.4	1	y7	0.278	0.040	71.257
113	FBLN1	P23142	FBLN1	Fibulin-1	TGYYFDGISR	589.8	2	694.4	1	y6	0.766	0.017	39.138
114	EFEMP1	Q12805	FBLN3	EGF-containing fibulin-like extracellular matrix protein 1	ADQVCINLR	544.8	2	675.4	1	y5	0.567	0.020	21.152
115	FCGR3A	P08637	FCG3A	Low affinity immunoglobulin gamma Fc region receptor III-A	AVVFLEPQWYR	704.4	2	749.4	1	y5	0.704	0.006	20.134
116	FCN3	O75636	FCN3	Ficolin-3	YGIDWASGR	512.7	2	576.3	1	y5	0.904	0.110	59.957
117	AFP	P02771	FETA	Alpha-fetoprotein	AENAVECFQTK	648.8	2	812.4	1	y6	0.540	0.058	46.215
118	AFP	P02771	FETA	Alpha-fetoprotein	GYQELLEK	490.3	2	759.4	1	y6	0.930	0.025	73.284
119	AHSG	P02765	FETUA	Alpha-2-HS-glycoprotein	EHAVEGDCDFQLLK	554.3	3	462.2	2	y7	0.550	0.119	3087.568
120	AHSG	P02765	FETUA	Alpha-2-HS-glycoprotein	FSVVYAK	407.2	2	579.4	1	y5	0.301	0.037	3517.634
121	FETUB	Q9UGM5	FETUB	Fetuin-B	IFFESVYGQCK	689.3	2	841.4	1	y7	0.958	0.071	88.538

122	FETUB	Q9UGM5	FETUB	Fetuin-B	LVVLPFPK	456.8	2	488.3	1	y4	0.175	0.026	22.163
123	CFHR2	P36980	FHR2	Complement factor H-related protein 2	ITCAEEGWSPTPK	738.3	2	529.3	1	y5	0.162	0.053	68.684
124	CFHR2	P36980	FHR2	Complement factor H-related protein 2	TGDIVEFVCK	584.3	2	781.4	1	y6	0.972	0.108	41.206
125	CFHR5	Q9BXR6	FHR5	Complement factor H-related protein 5	TGDAVEFQCK	577.8	2	711.3	1	y5	0.546	0.033	8.228
126	FGA	P02671	FIBA	Fibrinogen alpha chain	GLIDEVNQDFTNR	760.9	2	894.4	1	y7	0.551	0.048	15.095
127	FGA	P02671	FIBA	Fibrinogen alpha chain	GSESGIFTNTK	570.8	2	867.5	1	y8	0.169	0.056	160.686
128	FGB	P02675	FIBB	Fibrinogen beta chain	SILENLR	422.7	2	644.4	1	y5	1.000	0.071	12.864
129	FGG	P02679	FIBG	Fibrinogen gamma chain	DNCCILDER	597.7	2	304.2	1	y2	0.867	0.035	12.647
130	FN1	P02751	FN1	Fibronectin	GEWTCIAYSQLR	742.4	2	503.3	1	y4	0.289	0.085	908.444
131	FN1	P02751	FN1	Fibronectin	HTSVQTTSSGSGPFTDVR	622.0	3	734.4	1	y6	0.382	0.013	1111.509
132	FN1	P02751	FN1	Fibronectin	WCGTTQNYDADQK	793.8	2	347.1	1	b2	0.903	0.060	496.329
133	FSTL1	Q12841	FSTL1	Follistatin-related protein 1	LSFQEFLK	506.3	2	811.4	1	y6	0.575	0.067	3.082
134	GPX3	P22352	GPX3	Glutathione peroxidase 3	FYTFLK	409.7	2	508.3	1	y4	0.871	0.072	113.543
135	HABP2	Q14520	HABP2	Hyaluronan-binding protein 2	FTCACPDQFK	637.3	2	794.4	1	y6	0.121	0.169	42.135
136	HABP2	Q14520	HABP2	Hyaluronan-binding protein 2	LIANTLCNSR	581.3	2	935.4	1	y8	0.996	0.022	109.303
137	HPX	P02790	HEMO	Hemopexin	LLQDEFPGIPSPLDAAVECH R	788.7	3	809.9	2	y15	0.116	0.006	5059.430
138	HPX	P02790	HEMO	Hemopexin	SGAQATWTELPWPHEK	613.3	3	510.3	1	y4	0.445	0.023	11093.820
139	HGFAC	Q04756	HGFA	Hepatocyte growth factor activator	LCNIEPDER	573.3	2	304.2	1	y2	0.122	0.199	49.009
140	HGFAC	Q04756	HGFA	Hepatocyte growth factor activator	TTDVTQTFGIEK	670.3	2	1137.6	1	y10	0.310	0.121	100.943
141	HGFAC	Q04756	HGFA	Hepatocyte growth factor activator	VANYVDWINDR	682.8	2	917.4	1	y7	0.922	0.036	69.066
142	HP	P00738	HPT	Haptoglobin	VVLHPNYSQVDIGLIK	599.0	3	658.4	1	y6	0.787	0.049	34.816
143	HP	P00738	HPT	Haptoglobin	VGYVSGWGR	490.8	2	562.3	1	y5	0.996	0.162	787.831
144	HPR	P00739	HPTR	Haptoglobin-related protein	VGYVSGWGQSDNFK	772.4	2	1125.5	1	y10	0.917	0.128	35.263
145	IGFBP2	P18065	IBP2	Insulin-like growth factor-binding protein 2	GECWCVNPNTGK	711.3	2	347.1	1	b3	0.971	0.160	18.278
146	IGFBP3	P17936	IBP3	Insulin-like growth factor-binding protein 3	ALAQCAPPPAVCAELVR	912.0	2	1208.6	1	y11	0.484	0.092	43.942
147	IGFBP3	P17936	IBP3	Insulin-like growth factor-binding protein 3	YGQPLPGYTTK	612.8	2	876.5	1	y8	0.283	0.072	38.934
148	SERPIN G1	P05155	IC1	Plasma protease C1 inhibitor	DFTCVHQALK	406.9	3	478.8	2	y8	0.137	0.050	1610.122
149	IGF2	P01344	IGF2	Insulin-like growth factor II	GIVEECCFR	585.3	2	500.2	2	y7	0.090	0.040	52.722
150	IGHG1	P01857	IGHG1	Ig gamma-1 chain C region	NQVSLTCLVK	581.3	2	820.5	1	y7	0.946	0.150	41.104
151	IGHG3	P01860	IGHG3	Ig gamma-3 chain C region	WYVDGVEVHNAK	708.8	2	350.1	1	b2	0.889	0.088	1131.340
152	IGJ	P01591	IGJ	Immunoglobulin J chain	IIVPLNNR	469.8	2	613.3	1	y5	0.240	0.077	28.405
153	IGJ	P01591	IGJ	Immunoglobulin J chain	IVLVDNK	400.7	2	588.3	1	y5	0.981	0.012	34.100
154	IGJ	P01591	IGJ	Immunoglobulin J chain	SSEDPNEDIVER	695.3	2	971.5	1	y8	0.810	0.029	79.781

155	IL1RAP	Q9NPH3	IL1AP	Interleukin-1 receptor accessory protein	LYIEYGIQR	577.8	2	765.4	1	y6	0.328	0.077	9.432
156	IL1RAP	Q9NPH3	IL1AP	Interleukin-1 receptor accessory protein	NEVWWTIDGK	624.3	2	905.5	1	y7	0.775	0.142	12.967
157	SERPIN A5	P05154	IPSP	Plasma serine protease inhibitor	AVVEVDESGTR	581.3	2	763.4	1	y7	0.574	0.061	4.245
158	ISLR	O14498	ISLR	Immunoglobulin superfamily containing leucine-rich repeat protein	ALPGTPVASSQPR	640.9	2	548.8	2	y11	0.242	0.152	1.490
159	ITIH1	P19827	ITIH1	Inter-alpha-trypsin inhibitor heavy chain H1	EVAFDLEIPK	580.8	2	714.4	1	y6	0.842	0.032	857.598
160	ITIH1	P19827	ITIH1	Inter-alpha-trypsin inhibitor heavy chain H1	LDAQASFLPK	545.3	2	662.4	1	y6	1.000	0.116	391.128
161	ITIH2	P19823	ITIH2	Inter-alpha-trypsin inhibitor heavy chain H2	IQPSGGTNINEALLR	528.3	3	472.3	1	y4	0.674	0.140	1928.873
162	ITIH2	P19823	ITIH2	Inter-alpha-trypsin inhibitor heavy chain H2	TEVNVLPGAK	514.3	2	372.2	1	y4	0.859	0.042	854.545
163	ITIH2	P19823	ITIH2	Inter-alpha-trypsin inhibitor heavy chain H2	TILDDLR	423.2	2	631.3	1	y5	0.417	0.086	202.254
164	ITIH3	Q06033	ITIH3	Inter-alpha-trypsin inhibitor heavy chain H3	EVSFDVELPK	581.8	2	934.5	1	y8	0.797	0.036	56.176
165	ITIH3	Q06033	ITIH3	Inter-alpha-trypsin inhibitor heavy chain H3	DYIFGNYIER	645.3	2	898.4	1	y7	0.644	0.054	230.610
166	ITIH4	Q14624	ITIH4	Inter-alpha-trypsin inhibitor heavy chain H4	ILDDLSPR	464.8	2	702.3	1	y6	0.117	0.048	749.197
167	ITIH4	Q14624	ITIH4	Inter-alpha-trypsin inhibitor heavy chain H4	GPDVLTATVSGK	572.8	2	776.5	1	y8	0.777	0.040	803.344
168	ITIH4	Q14624	ITIH4	Inter-alpha-trypsin inhibitor heavy chain H4	NVVFVIDK	467.3	2	720.4	1	y6	0.992	0.076	1371.453
169	ITIH4	Q14624	ITIH4	Inter-alpha-trypsin inhibitor heavy chain H4	AGFSWIEVTFK	642.8	2	736.4	1	y6	0.277	0.035	1471.250
170	SERPIN A4	P29622	KAIN	Kallistatin	FYYLIASETPGK	694.9	2	301.2	1	y3	0.477	0.019	156.488
171	SERPIN A4	P29622	KAIN	Kallistatin	VGSALFLSHNLK	429.2	3	858.5	1	y7	0.784	0.044	79.114
172	SERPIN A4	P29622	KAIN	Kallistatin	FSISGSYVLDQILPR	848.0	2	385.3	1	y3	0.999	0.028	266.646
173	SERPIN A4	P29622	KAIN	Kallistatin	FFSAQTNR	485.7	2	676.3	1	y6	0.425	0.010	76.159
174	SERPIN A4	P29622	KAIN	Kallistatin	LGFTDLFSK	514.3	2	429.2	2	y7	0.809	0.059	80.901
175	SERPIN A4	P29622	KAIN	Kallistatin	WADLSGITK	495.8	2	733.4	1	y7	0.221	0.016	109.644
176	KLKB1	P03952	KLKB1	Plasma kallikrein	DSVTGTLPK	459.3	2	616.4	1	y6	0.312	0.026	89.136
177	KLKB1	P03952	KLKB1	Plasma kallikrein	VLTPDAFVCR	589.3	2	483.2	2	y8	0.564	0.037	190.244
178	LBP	P18428	LBP	Lipopolysaccharide-binding protein	ITLPDFTGDLR	624.3	2	920.4	1	y8	0.937	0.036	32.443
179	LCAT	P04180	LCAT	Phosphatidylcholine-sterol acyltransferase	SSGLVSNAPGVQIR	692.9	2	669.4	1	y6	0.864	0.061	33.162
180	LCAT	P04180	LCAT	Phosphatidylcholine-sterol acyltransferase	STELCGLWQGR	653.8	2	876.4	1	y7	0.428	0.071	33.397
181	LGALS3 BP	Q08380	LG3BP	Galectin-3-binding protein	ELSEALGQIFDSQR	796.9	2	950.5	1	y8	0.410	0.034	67.773
182	LGALS3 BP	Q08380	LG3BP	Galectin-3-binding protein	SDLAVPSELALLK	678.4	2	870.5	1	y8	0.077	0.099	29.630

183	LUM	P51884	LUM	Lumican	LPSGLPVSLLTLYLDNNK	653.0	3	766.4	1	y6	0.946	0.023	437.041
184	LUM	P51884	LUM	Lumican	ILGPLSYSK	489.3	2	751.4	1	y7	0.985	0.049	258.908
185	LYZ	P61626	LYSC	Lysozyme C	AWVAWR	394.7	2	531.3	1	y4	0.555	0.060	42.219
186	MBL2	P11226	MBL2	Mannose-binding protein C	FQASVATPR	488.8	2	701.4	1	y7	0.240	0.027	27.948
187	LCN2	P80188	NGAL	Neutrophil gelatinase-associated lipocalin	TFVPGCQPGEFTLGNIK	933.0	2	759.4	2	y14	0.072	0.135	7.935
188	NRP1	O14786	NRP1	Neuropilin-1	LYQVIFEGEIGK	698.4	2	779.4	1	y7	0.124	0.193	73.223
189	NRP1	O14786	NRP1	Neuropilin-1	GIIIQGGK	393.2	2	615.4	1	y6	0.176	0.108	7.919
190	PAPPA	Q13219	PAPP1	Pappalysin-1	LDGSTHLNIFFAK	731.9	2	495.3	2	y8	0.159	0.138	195.815
191	PGLYRP2	Q96PD5	PGRP2	N-acetylmuramoyl-L-alanine amidase	DTLPSCAVR	509.8	2	689.3	1	y6	0.799	0.011	213.035
192	PGLYRP2	Q96PD5	PGRP2	N-acetylmuramoyl-L-alanine amidase	TDCPGDALFDLLR	746.9	2	638.8	2	y11	0.740	0.031	214.725
193	GPLD1	P80108	PHLD	Phosphatidylinositol-glycan-specific phospholipase D	VAFLTVTLHQGGATR	785.9	2	726.4	1	y7	0.922	0.085	48.147
194	GPLD1	P80108	PHLD	Phosphatidylinositol-glycan-specific phospholipase D	IADVTSGLIGGEDGR	730.4	2	300.2	1	b3	0.551	0.053	48.445
195	PLGLA	Q15195	PLGA	Plasminogen-related protein A	DVVLFEK	425.2	2	635.4	1	y5	0.120	0.098	80.089
196	PLG	P00747	PLMN	Plasminogen	LSSPAVITDK	515.8	2	415.7	2	y8	0.802	0.017	631.677
197	PLG	P00747	PLMN	Plasminogen	EAQLPVIENK	570.8	2	699.4	1	y6	0.478	0.002	554.825
198	PON1	P27169	PON1	Serum paraoxonase/arylesterase 1	EVQPVELPNCNLVK	819.9	2	844.4	1	y7	0.867	0.071	1165.399
199	PON1	P27169	PON1	Serum paraoxonase/arylesterase 1	YVYIAELLAHK	660.4	2	781.5	1	y7	0.736	0.008	199.822
200	PON1	P27169	PON1	Serum paraoxonase/arylesterase 1	IQNILTEEPK	592.8	2	943.5	1	y8	0.751	0.041	563.370
201	POSTN	Q15063	POSTN	Periostin	GFEPGVTNILK	587.8	2	841.5	1	y8	0.790	0.187	4.757
202	PROS1	P07225	PROS	Vitamin K-dependent protein S	SFQTGLFTAAR	599.8	2	836.5	1	y8	1.000	0.077	39.223
203	PROS1	P07225	PROS	Vitamin K-dependent protein S	FSAEFDFR	509.7	2	784.4	1	y6	0.579	0.061	28.213
204	PROS1	P07225	PROS	Vitamin K-dependent protein S	NNLELSTPLK	564.8	2	787.5	1	y7	0.925	0.062	50.616
205	PROZ	P22891	PROZ	Vitamin K-dependent protein Z	ENFVLTTAK	511.8	2	533.3	1	y5	0.434	0.015	12.630
206	PVR	P15151	PVR	Poliovirus receptor	SVDIWLR	444.8	2	702.4	1	y5	0.101	0.041	10.037
207	QSOX1	O00391	QSOX1	Sulfhydryl oxidase 1	SFYTAYLQR	574.8	2	914.5	1	y7	0.988	0.134	9.125
208	REG3A	Q06141	REG3A	Regenerating islet-derived protein 3-alpha	NPSTISSPGHCASLSR	557.6	3	492.7	2	y9	0.105	0.134	5.053
209	RBP4	P02753	RET4	Retinol-binding protein 4	DPNGLPPEAQK	583.3	2	669.4	1	y6	0.282	0.013	654.191
210	RBP4	P02753	RET4	Retinol-binding protein 4	LIVHNGYCDGR	652.3	2	489.7	2	y8	0.461	0.016	946.375
211	APCS	P02743	SAMP	Serum amyloid P-component	AYSLFSYNTQGR	703.8	2	825.4	1	y7	0.936	0.147	858.901
212	APCS	P02743	SAMP	Serum amyloid P-component	IVLGQEQDSYGGK	697.4	2	1068.5	1	y10	0.869	0.026	109.438
213	APCS	P02743	SAMP	Serum amyloid P-component	AYSDLSR	406.2	2	577.3	1	y5	0.301	0.019	372.579
214	APCS	P02743	SAMP	Serum amyloid P-component	VGEYSLYIGR	578.8	2	708.4	1	y6	0.861	0.038	529.194

215	SEPP1	P49908	SEPP1	Selenoprotein P	LPTDSELAPR	549.8	2	493.3	2	y9	0.459	0.024	40.879
216	SHBG	P04278	SHBG	Sex hormone-binding globulin	TSSSFEVR	456.7	2	637.3	1	y5	0.461	0.042	44.265
217	SHBG	P04278	SHBG	Sex hormone-binding globulin	IALGGLLFPASNLR	721.4	2	657.4	1	y6	0.795	0.034	53.497
218	SOD3	P08294	SODE	Extracellular superoxide dismutase [Cu-Zn]	AGLAASLAGPHSIVGR	492.9	3	582.8	2	y12	0.466	0.155	4.875
219	SPARC	P09486	SPRC	SPARC	YIPPCLDSELTEFPLR	975.5	2	837.4	2	y14	0.138	0.065	67.553
220	SERPIN A7	P05543	THBG	Thyroxine-binding globulin	NALALFVLPK	543.3	2	787.5	1	y7	0.279	0.035	388.862
221	SERPIN A7	P05543	THBG	Thyroxine-binding globulin	GWVDLFVPK	530.8	2	817.5	1	y7	0.963	0.018	179.855
222	F2	P00734	THRB	Prothrombin	SGIECQLWR	574.8	2	762.4	1	y5	0.386	0.076	725.204
223	F2	P00734	THRB	Prothrombin	HQDFNSAVQLVENFCR	655.3	3	570.8	2	b10	0.157	0.094	1944.049
224	TF	P02787	TRFE	Serotransferrin	EGYYGYTGAFR	642.3	2	771.4	1	y7	0.833	0.177	7.982
225	GC	P02774	VTDB	Vitamin D-binding protein	HLSLLTTLSNR	418.9	3	376.2	1	y3	0.429	0.061	3597.870
226	GC	P02774	VTDB	Vitamin D-binding protein	VLEPTLK	400.2	2	587.3	1	y5	0.310	0.041	1313.944
227	VTN	P04004	VTNC	Vitronectin	FEDGVLDPDYPR	711.8	2	647.3	1	y5	0.993	0.053	414.913
228	AZGP1	P25311	ZA2G	Zinc-alpha-2-glycoprotein	YSLTYIYTGLSK	704.9	2	944.5	1	y8	0.301	0.027	570.526
229	AZGP1	P25311	ZA2G	Zinc-alpha-2-glycoprotein	SSGAFWK	391.7	2	608.3	1	y5	0.851	0.034	220.314
230	SERPIN A10	Q9UK55	ZPI	Protein Z-dependent protease inhibitor	LFDEINPETK	603.3	2	945.5	1	y8	0.794	0.023	9.542
231	SERPIN A10	Q9UK55	ZPI	Protein Z-dependent protease inhibitor	LILVDYILFK	618.9	2	1010.6	1	y8	0.645	0.013	35.525

Supplementary Table S2. List of 124 proteins that changed significantly in HCC compared with LC and Recovery patients in the training and test sets.

LC, liver cirrhosis; HCC, hepatocellular carcinoma; Recovery, Recovered from HCC.

N.	Gene Symbol	Uniprot Accession Number	Uniprot ID	Protein Name	Training set				Test set			
					LC vs. HCC		Recovery vs. HCC		LC vs. HCC		Recovery vs. HCC	
					Log2 (fold change)	Adjusted *P*	Log2 (fold change)	Adjusted *P*	Log2 (fold change)	Adjusted *P*	Log2 (fold change)	Adjusted *P*
1	SFN	P31947	1433S	14-3-3 protein sigma	-3.64E-01	1.07E-15	2.10E-01	8.58E-07	1.80E-01	2.38E-01	2.16E-02	8.97E-01
2	SERPINA1	P01009	A1AT	Alpha-1-antitrypsin	-4.43E-01	4.59E-15	-9.35E-02	7.51E-02	-2.42E-02	9.42E-01	1.23E+00	3.97E-05
3	SERPINF2	P08697	A2AP	Alpha-2-antiplasmin	4.93E-01	1.00E-20	-1.27E-01	1.97E-11	-1.02E-01	1.25E-03	-2.28E-01	4.41E-13
4	LRG1	P02750	A2GL	Leucine-rich alpha-2-glycoprotein	-2.93E-01	1.00E-20	-2.26E-02	9.05E-02	1.80E-01	1.11E-08	1.26E-01	5.17E-05
5	A2M	P01023	A2MG	Alpha-2-macroglobulin	2.31E-02	1.69E-01	1.55E-01	1.00E-20	-2.16E-01	1.57E-12	-2.13E-02	5.24E-01
6	SERPINA3	P01011	AACT	Alpha-1-antichymotrypsin	-7.54E-02	5.52E-04	-1.94E-01	1.00E-20	7.17E-02	1.43E-01	-3.03E-03	9.54E-01
7	IGFALS	P35858	ALS	Insulin-like growth factor-binding protein complex acid labile subunit	5.39E-01	1.00E-20	-3.64E-01	1.00E-20	3.61E-01	1.00E-20	-5.18E-01	1.00E-20
8	AMBP	P02760	AMBP	Protein AMBP	3.77E-01	1.00E-20	-1.52E-01	6.32E-06	2.05E-01	1.52E-03	-2.66E-01	2.21E-05
9	AGT	P01019	ANGT	Angiotensinogen	1.52E-01	1.30E-07	-3.97E-01	1.00E-20	5.93E-02	2.75E-01	-2.48E-01	5.90E-07
10	SERPINC1	P01008	ANT3	Antithrombin-III	8.43E-02	2.44E-06	-8.58E-02	1.69E-06	8.92E-02	1.97E-02	-2.75E-01	2.35E-13
11	APOA1	P02647	APOA1	Apolipoprotein A-I	-1.23E-01	6.07E-04	1.51E-01	2.65E-05	-3.73E-02	9.33E-02	-2.28E-01	1.00E-20
12	APOA4	P06727	APOA4	Apolipoprotein A-IV	-5.45E-01	1.00E-20	1.78E-01	1.00E-20	-2.25E-01	7.78E-07	7.50E-02	1.06E-01
13	APOC1	P02654	APOC1	Apolipoprotein C-I	4.42E-01	1.00E-20	-2.44E-01	1.00E-20	1.06E-01	2.91E-02	-4.59E-01	1.00E-20
14	APOC2	P02655	APOC2	Apolipoprotein C-II	9.28E-01	1.00E-20	-6.10E-01	1.00E-20	3.25E-01	1.00E-20	-6.62E-01	1.00E-20
15	APOC3	P02656	APOC3	Apolipoprotein C-III	1.79E-01	1.00E-20	-2.57E-01	1.00E-20	1.50E-01	2.92E-04	-6.41E-01	1.00E-20
16	APOC4	P55056	APOC4	Apolipoprotein C-IV	8.52E-01	1.00E-20	-7.65E-01	1.00E-20	3.97E-01	6.17E-10	-7.00E-01	1.00E-20
17	APOE	P02649	APOE	Apolipoprotein E	-6.54E-02	8.98E-08	6.44E-02	1.47E-07	-5.72E-02	4.14E-03	-1.16E-02	5.68E-01
18	APOF	Q13790	APOF	Apolipoprotein F	4.70E-01	1.00E-20	-2.23E-01	1.72E-15	2.54E-01	7.36E-02	-4.42E-01	8.32E-04
19	APOH	P02749	APOH	Beta-2-glycoprotein 1	3.80E-01	1.58E-06	-1.03E-01	1.90E-01	-1.68E-01	1.56E-01	9.60E-03	9.48E-01
20	APOL1	O14791	APOL1	Apolipoprotein L1	5.90E-01	1.00E-20	-4.14E-01	1.00E-20	8.82E-02	5.52E-01	-3.54E-01	5.89E-03
21	APOM	O95445	APOM	Apolipoprotein M	4.36E-01	1.00E-20	-1.76E-01	1.00E-20	1.67E-01	1.00E-20	-3.41E-01	1.00E-20
22	TGFBI	Q15582	BGH3	Transforming growth factor-beta-induced protein ig-h3	-4.97E-03	7.80E-01	-4.01E-02	2.95E-02	-1.49E-01	9.00E-02	5.20E-03	9.54E-01
23	BTD	P43251	BTD	Biotinidase	-1.61E-02	3.82E-01	-8.18E-02	8.06E-06	-4.63E-03	9.25E-01	-1.72E-01	2.09E-05
24	CD163	Q86VB7	C163A	Scavenger receptor cysteine-rich type 1 protein M130	-8.90E-01	1.00E-20	3.50E-01	3.06E-04	-2.15E-01	1.79E-01	3.98E-01	7.45E-03
25	C1QB	P02746	C1QB	Complement C1q subcomponent subunit B	-4.27E-01	1.00E-20	1.52E-01	2.74E-08	-2.14E-01	3.34E-05	1.43E-01	4.99E-03
26	C1QC	P02747	C1QC	Complement C1q subcomponent subunit C	-3.70E-01	1.00E-20	1.52E-01	1.54E-11	-1.95E-01	2.46E-04	8.28E-02	1.24E-01
27	C1RL	Q9NZP8	C1RL	Complement C1r subcomponent-like protein	7.60E-01	5.11E-05	-5.37E-01	4.23E-03	-3.34E-02	8.99E-01	1.54E-01	5.03E-01

28	C4BPA	P04003	C4BPA	C4b-binding protein alpha chain	4.77E-01	1.00E-20	-4.98E-01	1.00E-20	1.93E-01	1.15E-10	-5.32E-02	8.40E-02
29	C4BPB	P20851	C4BPB	C4b-binding protein beta chain	4.65E-01	1.00E-20	-3.21E-01	3.54E-13	3.55E-01	1.97E-02	-9.57E-02	5.44E-01
30	CTSB	P07858	CATB	Cathepsin B	-1.14E+00	1.00E-20	3.50E-01	3.37E-13	-1.60E-01	2.50E-01	9.59E-02	5.02E-01
31	CPB2	Q96IY4	CBPB2	Carboxypeptidase B2	2.60E-01	1.00E-20	-3.63E-01	1.00E-20	6.21E-02	1.41E-01	-2.72E-01	8.16E-12
32	CD5L	O43866	CD5L	CD5 antigen-like	-1.22E+00	1.00E-20	5.88E-01	1.00E-20	-6.87E-01	1.00E-20	4.77E-01	1.00E-20
33	CETP	P11597	CETP	Cholesteryl ester transfer protein	1.11E-01	5.50E-05	1.88E-01	5.58E-11	-1.39E-01	2.21E-01	6.65E-02	5.45E-01
34	CFH	P08603	CFAH	Complement factor H	-1.10E-02	6.88E-01	-7.99E-02	3.98E-03	1.36E-01	6.42E-02	-4.35E-02	5.45E-01
35	CFI	P05156	CFAI	Complement factor I	2.07E-01	1.00E-20	-1.99E-01	4.37E-16	1.64E-01	1.42E-02	-7.30E-02	2.84E-01
36	CHI3L1	P36222	CH3L1	Chitinase-3-like protein 1	-1.42E+00	1.00E-20	4.07E-01	4.23E-09	-5.25E-02	5.76E-01	5.03E-01	8.45E-10
37	BCHE	P06276	CHLE	Cholinesterase	7.31E-01	1.00E-20	-3.24E-01	1.00E-20	2.11E-01	1.00E-20	-5.73E-01	1.00E-20
38	CLU	P10909	CLUS	Clusterin	4.16E-01	1.00E-20	-2.06E-01	1.00E-20	7.59E-02	2.60E-04	-2.26E-01	1.00E-20
39	CNDP1	Q96KN2	CNDP1	Beta-Ala-His dipeptidase	1.65E-01	2.98E-04	-8.44E-01	1.00E-20	2.05E-01	6.51E-04	-5.18E-01	1.00E-20
40	C2	P06681	CO2	Complement C2	-9.86E-02	2.88E-08	5.24E-02	2.41E-03	-1.52E-02	8.66E-01	-3.33E-02	6.70E-01
41	C4A	P0C0L4	CO4A	Complement C4-A	3.33E-01	1.07E-15	-2.65E-01	1.30E-10	4.02E-02	5.52E-01	-2.86E-01	6.35E-07
42	C4B	P0C0L5	CO4B	Complement C4-B	-4.22E-01	1.00E-20	3.91E-01	1.00E-20	1.37E-01	4.92E-01	-1.52E-01	4.24E-01
43	C5	P01031	CO5	Complement C5	6.60E-02	5.76E-04	-1.89E-01	1.00E-20	9.23E-02	1.52E-03	-1.26E-01	8.67E-06
44	C6	P13671	CO6	Complement component C6	8.54E-02	5.69E-06	-2.23E-01	1.00E-20	1.67E-01	6.23E-10	-1.40E-01	1.02E-07
45	C7	P10643	CO7	Complement component C7	-1.05E+00	1.00E-20	2.65E-01	5.38E-12	-1.65E-01	2.81E-07	2.80E-01	1.00E-20
46	C8B	P07358	CO8B	Complement component C8 beta chain	1.26E-01	8.12E-11	-1.52E-01	6.26E-15	2.95E-02	4.12E-01	-2.03E-01	1.08E-10
47	COLEC11	Q9BWP8	COL11	Collectin-11	-5.56E-02	2.09E-01	-6.41E-02	1.50E-01	-1.88E-01	6.03E-02	1.67E-01	8.40E-02
48	CPN2	P22792	CPN2	Carboxypeptidase N subunit 2	3.45E-01	1.00E-20	-1.93E-01	1.00E-20	8.66E-02	5.36E-03	-1.94E-01	2.35E-10
49	CRTAC1	Q9NQ79	CRAC1	Cartilage acidic protein 1	-4.07E-02	9.02E-02	1.06E-01	1.29E-05	-8.56E-02	4.44E-01	1.64E-02	8.93E-01
50	CRP	P02741	CRP	C-reactive protein	-8.61E-01	1.00E-20	-5.26E-01	1.00E-20	5.85E-01	8.63E-02	5.68E-01	8.40E-02
51	PPBP	P02775	CXCL7	Platelet basic protein	7.54E-01	1.00E-20	-8.33E-01	1.00E-20	2.11E-01	1.00E-20	-1.61E-01	8.56E-12
52	F10	P00742	FA10	Coagulation factor X	-1.29E-01	1.08E-02	-1.99E-01	1.04E-04	5.43E-02	5.52E-01	-1.31E-01	1.13E-01
53	F11	P03951	FA11	Coagulation factor XI	6.36E-01	1.00E-20	-5.92E-01	1.00E-20	5.15E-01	1.00E-20	-5.90E-01	1.00E-20
54	F12	P00748	FA12	Coagulation factor XII	2.40E-01	1.00E-20	-1.70E-01	9.86E-15	-2.51E-02	3.53E-01	-1.72E-02	5.16E-01
55	F9	P00740	FA9	Coagulation factor IX	5.45E-02	2.18E-02	-3.03E-01	1.00E-20	1.97E-01	1.04E-09	-2.84E-01	1.00E-20
56	FBLN1	P23142	FBLN1	Fibulin-1	-6.60E-01	1.00E-20	3.70E-01	1.00E-20	-2.98E-01	1.00E-20	2.69E-01	1.00E-20
57	EFEMP1	Q12805	FBLN3	EGF-containing fibulin-like extracellular matrix protein 1	-1.15E+00	1.00E-20	5.42E-01	1.00E-20	-3.88E-01	2.97E-03	5.47E-01	1.51E-05
58	FCGR3A	P08637	FCG3A	Low affinity immunoglobulin gamma Fc region receptor III-A	-6.91E-01	1.00E-20	3.49E-01	1.00E-20	-1.16E-01	5.41E-01	3.67E-01	2.50E-02
59	FCN3	O75636	FCN3	Ficolin-3	6.68E-01	1.00E-20	-6.15E-02	1.39E-01	7.87E-03	9.49E-01	-1.68E-01	2.29E-01
60	AFP	P02771	FETA	Alpha-fetoprotein	6.62E-01	3.39E-04	-7.44E-01	6.13E-05	9.78E-01	1.12E-06	-4.71E-01	2.07E-02
61	AHSG	P02765	FETUA	Alpha-2-HS-glycoprotein	3.71E-01	1.00E-20	6.46E-03	8.02E-01	-6.83E-02	6.03E-02	-1.73E-01	3.75E-07
62	FETUB	Q9UGM5	FETUB	Fetuin-B	1.18E-01	1.17E-01	-1.08E-01	1.50E-01	9.01E-02	4.51E-02	-1.26E-01	2.71E-03

63	CFHR2	P36980	FHR2	Complement factor H-related protein 2	8.04E-02	1.90E-03	-2.57E-01	1.00E-20	3.28E-01	1.00E-20	-1.06E-01	4.45E-04
64	CFHR5	Q9BXR6	FHR5	Complement factor H-related protein 5	6.11E-02	4.26E-02	-1.61E-01	2.04E-07	3.20E-01	3.11E-03	-8.98E-02	4.45E-01
65	FGA	P02671	FIBA	Fibrinogen alpha chain	-1.06E+00	1.00E-20	1.31E+00	1.00E-20	-1.37E-01	8.81E-02	7.13E-01	1.00E-20
66	FGB	P02675	FIBB	Fibrinogen beta chain	-1.87E+00	1.00E-20	1.71E+00	1.00E-20	7.88E-02	7.52E-01	8.58E-01	2.67E-05
67	FGG	P02679	FIBG	Fibrinogen gamma chain	-1.69E+00	1.00E-20	1.81E+00	1.00E-20	-5.66E-02	8.35E-01	9.86E-01	2.27E-06
68	FN1	P02751	FN1	Fibronectin	1.24E+00	1.00E-20	-9.23E-01	1.00E-20	1.44E+00	1.00E-20	-1.50E+00	1.00E-20
69	FSTL1	Q12841	FSTL1	Follistatin-related protein 1	-4.24E-01	1.00E-20	-5.05E-03	9.13E-01	-1.23E-01	1.11E-01	1.07E-01	1.55E-01
70	GPX3	P22352	GPX3	Glutathione peroxidase 3	-3.64E-01	1.00E-20	6.45E-02	3.56E-02	-4.07E-02	7.14E-01	-4.42E-02	6.46E-01
71	HABP2	Q14520	HABP2	Hyaluronan-binding protein 2	-2.66E-02	2.09E-01	-2.01E-01	1.00E-20	2.42E-01	5.36E-03	-5.44E-02	5.45E-01
72	HPX	P02790	HEMO	Hemopexin	6.66E-01	1.00E-20	-3.19E-01	1.00E-20	1.41E-01	8.74E-10	-2.84E-01	1.00E-20
73	HGFAC	Q04756	HGFA	Hepatocyte growth factor activator	1.70E-01	9.14E-08	-2.52E-01	4.66E-15	2.01E-01	2.47E-05	-2.97E-01	1.79E-10
74	HP	P00738	HPT	Haptoglobin	8.54E-01	1.00E-20	-1.26E+00	1.00E-20	-1.10E-01	1.33E-01	4.28E-01	3.73E-10
75	HPR	P00739	HPTR	Haptoglobin-related protein	5.62E-01	1.33E-06	-7.47E-01	3.68E-10	-1.21E-01	6.82E-01	1.53E-01	5.45E-01
76	IGFBP2	P18065	IBP2	Insulin-like growth factor-binding protein 2	-7.94E-01	1.00E-20	3.96E-01	1.01E-07	-1.97E-01	1.93E-01	3.66E-01	9.01E-03
77	IGFBP3	P17936	IBP3	Insulin-like growth factor-binding protein 3	6.09E-01	1.00E-20	-4.31E-01	1.00E-20	3.19E-01	1.00E-20	-4.14E-01	1.00E-20
78	SERPING1	P05155	IC1	Plasma protease C1 inhibitor	-1.36E-01	1.89E-12	-9.38E-02	5.21E-07	-1.16E-01	2.79E-01	-3.27E-02	7.62E-01
79	IGF2	P01344	IGF2	Insulin-like growth factor II	4.62E-01	1.00E-20	-3.03E-01	1.00E-20	2.94E-01	4.26E-03	-3.36E-01	7.46E-04
80	IGHG1	P01857	IGHG1	Ig gamma-1 chain C region	-1.19E+00	1.00E-20	5.98E-01	1.00E-20	-6.18E-01	6.12E-02	1.02E+00	8.74E-04
81	IGHG3	P01860	IGHG3	Ig gamma-3 chain C region	-1.30E+00	1.00E-20	6.79E-01	2.94E-12	-6.12E-01	7.21E-02	9.91E-01	1.83E-03
82	IGJ	P01591	IGJ	Immunoglobulin J chain	-6.50E-01	1.00E-20	1.21E-01	1.02E-01	-6.62E-01	1.00E-20	7.15E-01	1.00E-20
83	IL1RAP	Q9NPH3	IL1AP	Interleukin-1 receptor accessory protein	-2.28E-01	1.03E-04	-8.26E-02	1.60E-01	-1.76E-01	2.47E-02	-9.40E-02	2.31E-01
84	SERPINA5	P05154	IPSP	Plasma serine protease inhibitor	-1.66E-01	4.21E-10	2.96E-02	2.42E-01	9.77E-02	7.23E-01	-6.19E-01	6.23E-03
85	ISLR	O14498	ISLR	Immunoglobulin superfamily containing leucine-rich repeat protein	-6.94E-01	1.00E-20	1.89E-01	3.03E-10	-4.14E-01	6.38E-05	3.55E-01	4.45E-04
86	ITIH1	P19827	ITIH1	Inter-alpha-trypsin inhibitor heavy chain H1	5.64E-01	1.00E-20	-3.23E-01	1.00E-20	1.71E-01	2.78E-08	-4.37E-01	1.00E-20
87	ITIH2	P19823	ITIH2	Inter-alpha-trypsin inhibitor heavy chain H2	3.17E-01	1.00E-20	-2.07E-01	1.00E-20	9.38E-02	3.43E-01	-3.15E-01	3.48E-04
88	ITIH3	Q06033	ITIH3	Inter-alpha-trypsin inhibitor heavy chain H3	-2.34E-01	1.00E-20	2.20E-01	1.00E-20	1.36E-01	8.00E-07	6.41E-03	8.40E-01
89	ITIH4	Q14624	ITIH4	Inter-alpha-trypsin inhibitor heavy chain H4	2.50E-01	1.00E-20	-1.09E-01	2.03E-04	3.64E-02	6.79E-01	-2.30E-03	9.72E-01
90	SERPINA4	P29622	KAIN	Kallistatin	3.59E-01	1.00E-20	-2.75E-01	1.00E-20	7.41E-02	4.92E-05	-4.26E-01	1.00E-20
91	KLKB1	P03952	KLKB1	Plasma kallikrein	4.65E-01	1.00E-20	-3.38E-01	1.00E-20	1.02E-01	2.60E-04	-3.30E-01	1.00E-20
92	LBP	P18428	LBP	Lipopolysaccharide-binding protein	-1.59E-01	1.89E-12	-1.89E-01	4.37E-16	5.32E-02	6.99E-01	8.78E-02	4.83E-01
93	LCAT	P04180	LCAT	Phosphatidylcholine-sterol acyltransferase	4.90E-01	3.29E-09	-8.50E-02	2.98E-01	1.89E-01	4.32E-08	-3.98E-01	1.00E-20
94	LGALS3BP	Q08380	LG3BP	Galectin-3-binding protein	-6.17E-01	1.00E-20	2.69E-01	1.00E-20	-4.09E-01	1.00E-20	3.11E-01	1.00E-20

95	LUM	P51884	LUM	Lumican	-5.70E-01	1.00E-20	3.57E-01	1.00E-20	-3.13E-01	1.00E-20	1.56E-01	2.05E-08
96	LYZ	P61626	LYSC	Lysozyme C	-1.87E-01	2.97E-06	-5.49E-02	1.60E-01	9.29E-02	3.62E-01	9.03E-02	3.72E-01
97	MBL2	P11226	MBL2	Mannose-binding protein C	-3.99E-01	1.00E-20	-1.79E-01	1.05E-10	-3.24E-01	1.56E-01	2.63E-01	2.41E-01
98	LCN2	P80188	NGAL	Neutrophil gelatinase-associated lipocalin	1.53E-01	4.92E-05	-1.70E-01	7.16E-06	1.02E-01	5.30E-01	9.83E-02	5.16E-01
99	NRP1	O14786	NRP1	Neuropilin-1	2.91E-01	1.87E-01	5.03E-01	2.30E-02	-2.98E-02	9.10E-01	-1.64E-01	4.91E-01
100	PAPPA	Q13219	PAPP1	Pappalysin-1	-2.61E-01	3.00E-06	1.25E-01	2.30E-02	-9.92E-03	9.42E-01	-2.00E-01	1.34E-01
101	PGLYRP2	Q96PD5	PGRP2	N-acetylmuramoyl-L-alanine amidase	7.34E-03	6.71E-01	3.34E-02	5.27E-02	-5.97E-02	1.06E-01	-1.78E-01	2.83E-07
102	GPLD1	P80108	PHLD	Phosphatidylinositol-glycan-specific phospholipase D	3.46E-01	2.08E-08	-2.62E-01	1.92E-05	2.20E-02	7.23E-01	-2.42E-01	2.86E-06
103	PLGLA	Q15195	PLGA	Plasminogen-related protein A	1.30E+00	1.00E-20	-4.08E-01	1.00E-20	1.46E-01	8.22E-02	-2.18E-01	5.55E-03
104	PLG	P00747	PLMN	Plasminogen	3.06E-01	1.00E-20	-3.14E-01	1.00E-20	1.41E-01	7.52E-12	-2.75E-01	1.00E-20
105	PON1	P27169	PON1	Serum paraoxonase/arylesterase 1	3.39E-01	1.00E-20	-2.70E-01	1.00E-20	1.10E-01	1.43E-04	-4.40E-01	1.00E-20
106	POSTN	Q15063	POSTN	Periostin	-3.80E-01	1.00E-20	2.40E-01	3.39E-09	-1.64E-01	1.53E-01	3.06E-01	4.16E-03
107	PROS1	P07225	PROS	Vitamin K-dependent protein S	-1.91E-01	3.25E-08	-3.14E-01	1.00E-20	3.53E-02	5.21E-01	-6.19E-02	2.07E-01
108	PROZ	P22891	PROZ	Vitamin K-dependent protein Z	-5.12E-01	1.00E-20	-3.03E-01	1.00E-20	2.90E-01	4.25E-02	-2.00E-01	1.51E-01
109	PVR	P15151	PVR	Poliovirus receptor	-5.42E-01	1.00E-20	-2.67E-02	4.36E-01	-1.21E-01	2.09E-01	1.64E-01	7.47E-02
110	QSOX1	O00391	QSOX1	Sulfhydryl oxidase 1	-5.47E-01	1.00E-20	1.41E-01	1.51E-03	-3.09E-01	1.98E-03	3.40E-01	4.45E-04
111	REG3A	Q06141	REG3A	Regenerating islet-derived protein 3-alpha	-4.59E-01	8.52E-07	4.20E-01	6.08E-06	1.71E-01	3.26E-01	2.93E-01	7.10E-02
112	RBP4	P02753	RET4	Retinol-binding protein 4	4.48E-01	1.00E-20	-5.28E-01	1.00E-20	6.59E-02	7.53E-01	-3.63E-01	4.34E-02
113	APCS	P02743	SAMP	Serum amyloid P-component	3.11E-01	3.71E-08	-5.23E-01	1.00E-20	1.64E-01	1.98E-03	-3.54E-01	3.25E-12
114	SEPP1	P49908	SEPP1	Selenoprotein P	2.66E-01	1.00E-20	-2.73E-01	1.00E-20	-2.19E-01	6.03E-02	-1.94E-01	8.40E-02
115	SHBG	P04278	SHBG	Sex hormone-binding globulin	7.88E-02	1.05E-01	3.76E-01	3.28E-14	1.61E-01	3.67E-15	-6.95E-02	3.52E-04
116	SOD3	P08294	SODE	Extracellular superoxide dismutase [Cu-Zn]	-4.82E-01	1.00E-20	3.30E-01	4.37E-16	-4.90E-02	8.39E-01	3.88E-01	4.97E-02
117	SPARC	P09486	SPRC	SPARC	6.96E-01	1.00E-20	-8.09E-01	1.00E-20	1.36E-01	2.46E-01	-9.55E-02	4.25E-01
118	SERPINA7	P05543	THBG	Thyroxine-binding globulin	1.21E-01	5.16E-01	5.13E-03	9.77E-01	8.42E-01	4.87E-07	-2.69E-01	1.14E-01
119	F2	P00734	THRB	Prothrombin	4.32E-01	1.00E-20	-2.60E-01	1.00E-20	1.84E-01	2.32E-04	-2.93E-01	2.19E-09
120	TF	P02787	TRFE	Serotransferrin	5.97E-01	1.00E-20	-4.19E-01	1.00E-20	-5.04E-01	6.00E-02	-2.04E-01	4.63E-01
121	GC	P02774	VTDB	Vitamin D-binding protein	3.26E-01	1.00E-20	-2.00E-01	1.00E-20	1.73E-01	2.78E-08	-2.12E-01	1.07E-11
122	VTN	P04004	VTNC	Vitronectin	3.33E-01	1.00E-20	-2.44E-01	1.00E-20	-3.20E-02	8.35E-01	-2.59E-01	3.48E-02
123	AZGP1	P25311	ZA2G	Zinc-alpha-2-glycoprotein	-2.88E-02	6.50E-02	-1.73E-01	1.00E-20	-4.99E-03	8.53E-01	-8.82E-02	4.72E-05
124	SERPINA10	Q9UK55	ZPI	Protein Z-dependent protease inhibitor	-1.09E-02	6.24E-01	-1.47E-01	4.02E-11	2.06E-01	1.10E-10	-2.81E-01	1.00E-20

Supplementary Table S3. Targets with AUROC values ≥ 0.800 in LC vs. HCC in the training set. Corresponding AUROC values in the test set are also presented.

AUROC, area under the receiver operating curve; LC, liver cirrhosis; HCC, hepatocellular carcinoma; Recovery, Recovered from HCC.

Number	Gene Symbol	Uniprot Accession Number	Uniprot ID	Protein Name	Peptide Sequence	Training set		Test set	
						LC vs. HCC	HCC vs. Recovery	LC vs. HCC	HCC vs. Recovery
						AUROC	AUROC	AUROC	AUROC
1	A2AP	P08697	A2AP	Alpha-2-antiplasmin	LCQDLGPGAFR	0.940	0.696	0.599	0.812
2					WFLLEQPEIQVAHFPFK	0.841	0.586	0.581	0.812
3	ALS	P35858	ALS	Insulin-like growth factor-binding protein complex acid labile subunit	NLIAAVAPGAFLGLK	0.826	0.655	0.756	0.815
4					LEYLLLSR	0.826	0.698	0.734	0.817
5					LEALPNSLLAPLGR	0.830	0.681	0.753	0.808
6	AMBP	P02760	AMBP	Protein AMBP	TVAACNLPIVR	0.851	0.655	0.720	0.760
7	APOC2	P02655	APOC2	Apolipoprotein C-II	ESLSSYWESAK	0.812	0.558	0.623	0.674
8					TYLPAVDEK	0.805	0.567	0.656	0.724
9	APOC4	P55056	APOC4	Apolipoprotein C-IV	AWFLESK	0.804	0.624	0.654	0.758
10	APOL1	O14791	APOL1	Apolipoprotein L1	LNILNNNYK	0.855	0.671	0.696	0.728
11	C4BPA	P04003	C4BPA	C4b-binding protein alpha chain	TWYPEVPK	0.800	0.706	0.608	0.617
12					LSLEIEQLELQR	0.801	0.714	0.608	0.622
13	CATB	P07858	CATB	Cathepsin B	EQWPQCPTIK	0.846	0.620	0.631	0.602
14	CBPB2	Q96IY4	CBPB2	Carboxypeptidase B2	YPLYVLK	0.839	0.717	0.648	0.711
15					DTGTYGFLLPER	0.811	0.723	0.715	0.765
16	CD5L	O43866	CD5L	CD5 antigen-like	NTCNHDEDTWVECEDPFDLR	0.865	0.574	0.718	0.658
17					DVAVLCR	0.826	0.589	0.709	0.654
18					LVGGDNLCSGR	0.882	0.572	0.725	0.653
19	CFAI	P05156	CFAI	Complement factor I	VFSLQWGEVK	0.848	0.728	0.695	0.606
20	CHLE	P06276	CHLE	Cholinesterase	IFFPGVSEFGK	0.879	0.646	0.719	0.821
21					YLTLNTESTR	0.877	0.630	0.720	0.824
22	CLUS	P10909	CLUS	Clusterin	EIQNAVNGVK	0.929	0.649	0.696	0.783
23					ASSIIDELFQDR	0.918	0.651	0.663	0.768
24	CO7	P10643	CO7	Complement component C7	LSGNVLSYTFQVK	0.822	0.593	0.629	0.644
25					DSCTLPASAEK	0.818	0.582	0.610	0.658
26	CPN2	P22792	CPN2	Carboxypeptidase N subunit 2	TLNLAQNLLAQLPEELFHPLTSLQTLK	0.910	0.646	0.682	0.706
27					LSNNALSGLPQGVFGK	0.868	0.661	0.674	0.688
28	CXCL7	P02775	CXCL7	Platelet basic protein	NIQSLEVIGK	0.861	0.630	0.617	0.620

29					GTHCNQVEVIATLK	0.852	0.625	0.633	0.631
30	FA11	P03951	FA11	Coagulation factor XI	DSVTETLPR	0.883	0.595	0.781	0.778
31					ALSGFSLQSCR	0.861	0.671	0.735	0.745
32	FBLN3	Q12805	FBLN3	EGF-containing fibulin-like extracellular matrix protein 1	ADQVCINLR	0.833	0.591	0.686	0.714
33	FN1	P02751	FN1	Fibronectin	GEWTCIAYSQLR	0.910	0.741	0.914	0.882
34					HTSVQTTSSGSGPFTDVR	0.895	0.729	0.918	0.890
35					WCGTTQNYDADQK	0.900	0.753	0.921	0.893
36	HEMO	P02790	HEMO	Hemopexin	SGAQATWTELPWPHEK	0.817	0.767	0.634	0.685
37	HGFA	Q04756	HGFA	Hepatocyte growth factor activator	TTDVTQTFGIEK	0.807	0.647	0.682	0.751
38	IBP3	P17936	IBP3	Insulin-like growth factor-binding protein 3	ALAQCAPPPAVCAELVR	0.856	0.703	0.791	0.848
39					YGQPLPGYTTK	0.845	0.705	0.740	0.815
40	IGF2	P01344	IGF2	Insulin-like growth factor II	GIVEECCFR	0.823	0.681	0.744	0.831
41	ITIH1	P19827	ITIH1	Inter-alpha-trypsin inhibitor heavy chain H1	EVAFDLEIPK	0.980	0.716	0.745	0.872
42					LDAQASFLPK	0.969	0.712	0.755	0.831
43	ITIH2	P19823	ITIH2	Inter-alpha-trypsin inhibitor heavy chain H2	TEVNVLPGAK	0.928	0.692	0.780	0.889
44					TILDDLR	0.825	0.681	0.760	0.809
45	ITIH4	Q14624	ITIH4	Inter-alpha-trypsin inhibitor heavy chain H4	AGFSWIEVTFK	0.819	0.722	0.567	0.587
46	KAIN	P29622	KAIN	Kallistatin	VGSALFLSHNLK	0.815	0.648	0.793	0.886
47					FSISGSYVLDQILPR	0.922	0.638	0.695	0.843
48					FFSAQTNR	0.869	0.656	0.708	0.845
49					LGFTDLFSK	0.893	0.673	0.696	0.796
50					WADLSGITK	0.881	0.625	0.722	0.833
51	KLKB1	P03952	KLKB1	Plasma kallikrein	DSVTGTLPK	0.852	0.607	0.642	0.813
52					VLTPDAFVCR	0.864	0.619	0.668	0.795
53	LCAT	P04180	LCAT	Phosphatidylcholine-sterol acyltransferase	SSGLVSNAPGVQIR	0.845	0.576	0.742	0.789
54					STELCGLWQGR	0.924	0.632	0.712	0.837
55	PHLD	P80108	PHLD	Phosphatidylinositol-glycan-specific phospholipase D	VAFLTVTLHQGGATR	0.812	0.624	0.605	0.678
56					IADVTSGLIGGEDGR	0.832	0.672	0.659	0.704
57	PLGA	Q15195	PLGA	Plasminogen-like protein A	DVVLFEK	0.928	0.698	0.711	0.739
58	PLMN	P00747	PLMN	Plasminogen	LSSPAVITDK	0.883	0.720	0.761	0.799
59					EAQLPVIENK	0.895	0.781	0.711	0.722
60	PON1	P27169	PON1	Serum paraoxonase/arylesterase 1	YVYIAELLAHK	0.808	0.623	0.673	0.748
61					IQNILTEEPK	0.829	0.617	0.677	0.714
62	SAMP	P02743	SAMP	Serum amyloid P-component	AYSLFSYNTQGR	0.822	0.642	0.627	0.683
63					IVLGQEQDSYGGK	0.831	0.664	0.649	0.686

64					VGEYSLYIGR	0.807	0.621	0.661	0.712
65	SPRC	P09486	SPRC	SPARC	YIPPCLDSELTEFPLR	0.848	0.642	0.626	0.610
66	THRB	P00734	THRB	Prothrombin	SGIECQLWR	0.956	0.712	0.803	0.889
67					HQDFNSAVQLVENFCR	0.967	0.743	0.815	0.918
68	VTDB	P02774	VTDB	Vitamin D-binding protein	HLSLLTTLSNR	0.862	0.652	0.678	0.790
69					VLEPTLK	0.888	0.648	0.703	0.763
70	VTNC	P04004	VTNC	Vitronectin	FEDGVLDPDYPR	0.879	0.669	0.663	0.783

Supplementary Table S4. Sample size calculation.

**Sample sizes of case / control**		**Type Ⅰ Error (α)**			
		0.20	0.10	0.05	0.01
**Type Ⅱ Error (β)**	0.20	7 / 7	10 / 10	13 / 13	20 / 20
	0.10	10 / 10	13 / 13	17 / 17	24 / 24
	0.05	12 / 12	16 / 16	**20 / 20**	28 / 28
	0.01	18 / 18	22 / 22	26 / 26	36 / 36

Supplementary Table S5. Additional analyses with male patient-only data.

Data set	Peptide Sequence		Male patient-only data		Whole data	
			LC vs. HCC	HCC vs. Recovery	LC vs. HCC	HCC vs. Recovery
MRM-MS (Training set)	GEWTCIAYSQLR	*P*-value	< 0.001	0.002	< 0.001	0.001
		AUROC (95% CI)	0.932 (0.812 - 0.986)	0.750 (0.604 - 0.864)	0.911 (0.809 - 0.969)	0.746 (0.617 - 0.849)
	HTSVQTTSSGSGPFTDVR	*P*-value	< 0.001	0.005	< 0.001	0.002
		AUROC (95% CI)	0.932 (0.812 - 0.986)	0.733 (0.585 - 0.850)	0.901 (0.796 - 0.963)	0.733 (0.603 - 0.839)
	WCGTTQNYDADQK	*P*-value	< 0.001	0.003	< 0.001	0.001
		AUROC (95% CI)	0.919 (0.794 - 0.980)	0.759 (0.614 - 0.870)	0.904 (0.801 - 0.965)	0.758 (0.630 - 0.859)
MRM-MS (Test set)	GEWTCIAYSQLR	*P*-value	0.047	< 0.001	< 0.001	< 0.001
		AUROC (95% CI)	0.929 (0.741 - 0.994)	0.844 (0.698 - 0.937)	0.916 (0.815 - 0.972)	0.881 (0.772 - 0.950)
	HTSVQTTSSGSGPFTDVR	*P*-value	0.047	< 0.001	< 0.001	< 0.001
		AUROC (95% CI)	0.929 (0.741 - 0.994)	0.855 (0.712 - 0.944)	0.920 (0.820 - 0.974)	0.889 (0.781 - 0.955)
	WCGTTQNYDADQK	*P*-value	0.032	< 0.001	< 0.001	< 0.001
		AUROC (95% CI)	0.952 (0.774 - 0.998)	0.868 (0.728 - 0.953)	0.926 (0.828 - 0.977)	0.896 (0.789 - 0.960)
ELISA (Validation set)		*P*-value	NA	0.019	< 0.001	0.011
		AUROC (95% CI)	NA	0.636 (0.459 - 0.789)	0.832 (0.673 - 0.935)	0.658 (0.489 - 0.802)

MRM-MS, multiple reaction monitoring-mass spectrometry; ELISA, enzyme-linked immunosorbent assay; LC, liver cirrhosis; HCC, hepatocellular carcinoma; Recovery, HCC patients who recovered; AUROC, area under the receiver operating characteristic curve; CI, confidence interval; NA, not applicable.

Supplementary Figure S1.

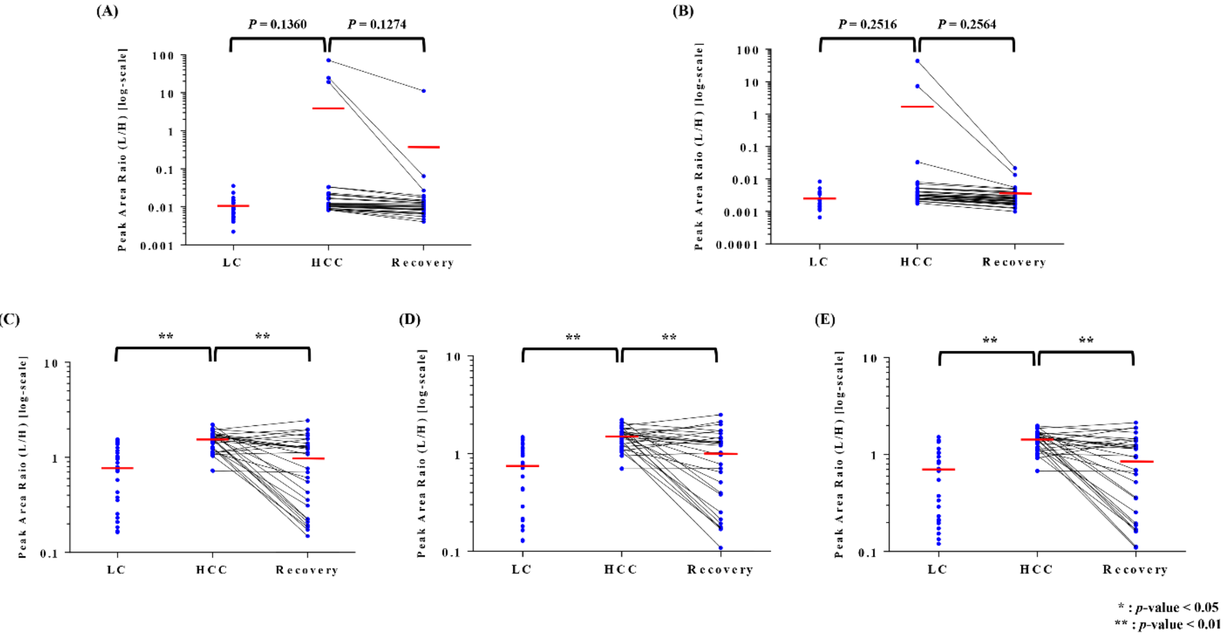

Supplementary Figure S2.

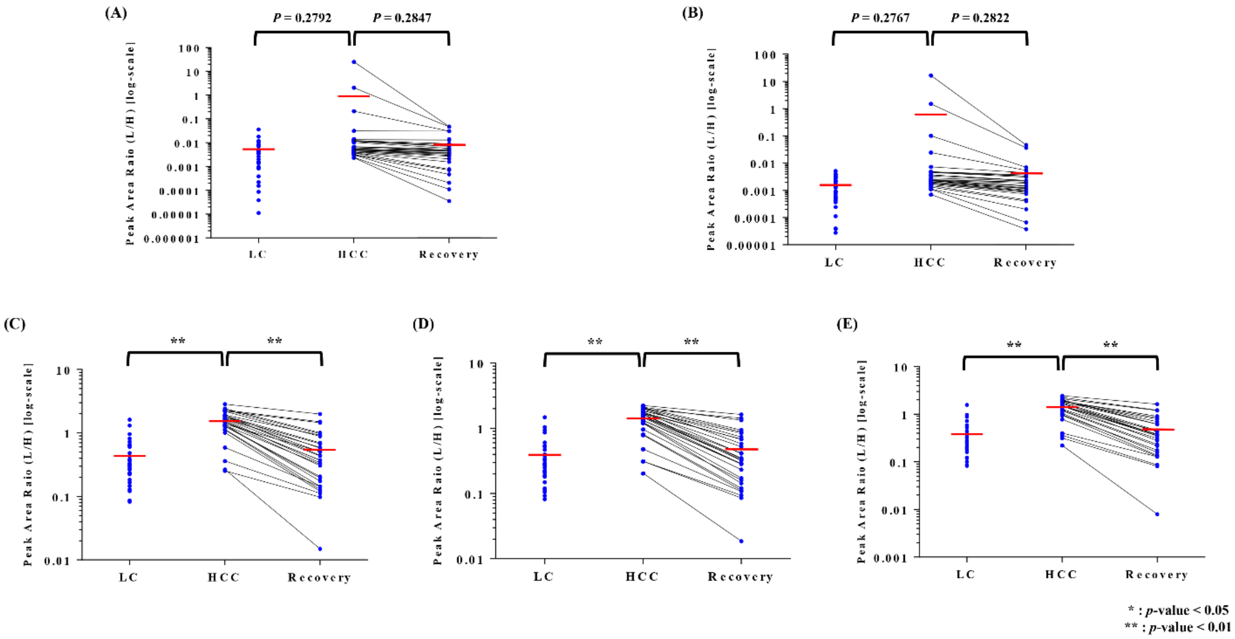

Supplementary Figure S3.

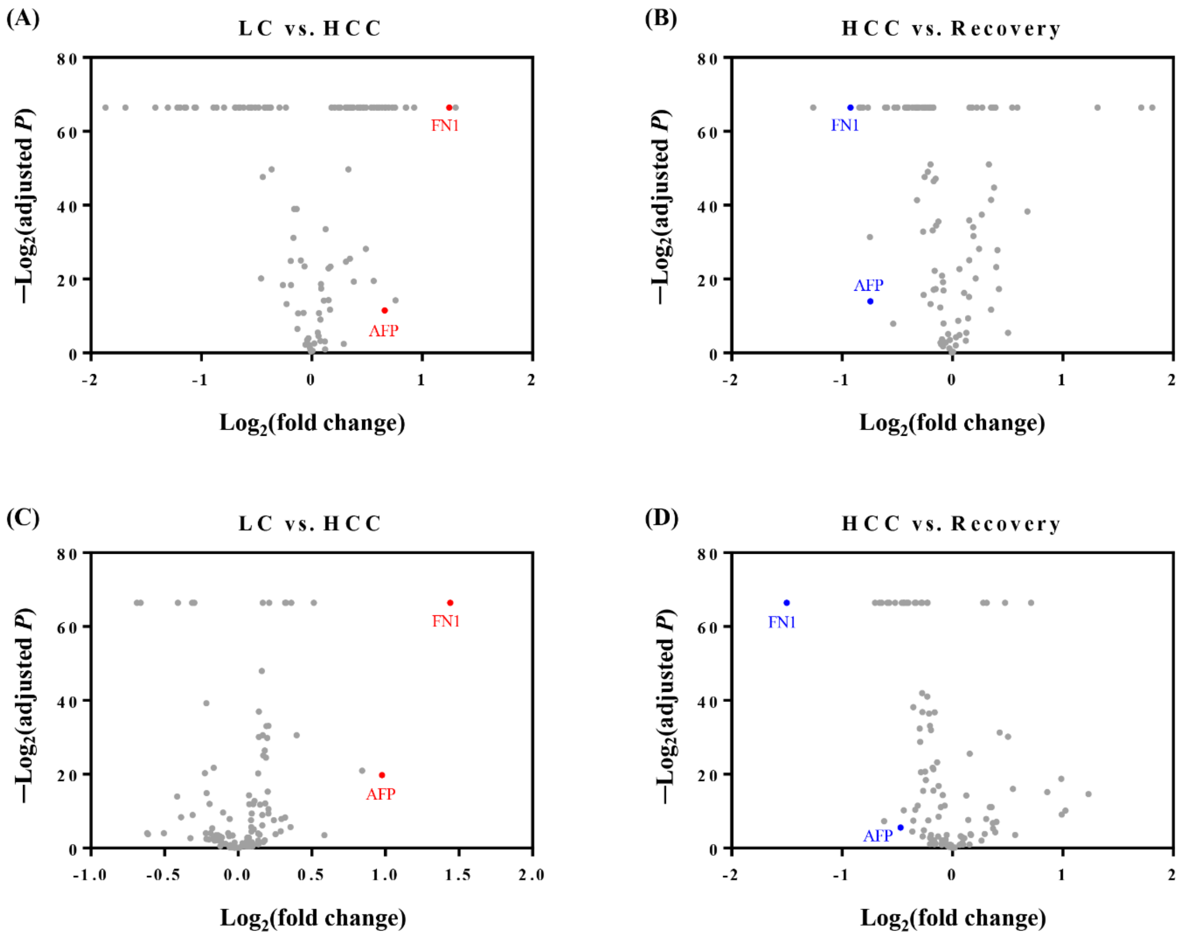

Supplementary Figure S4.

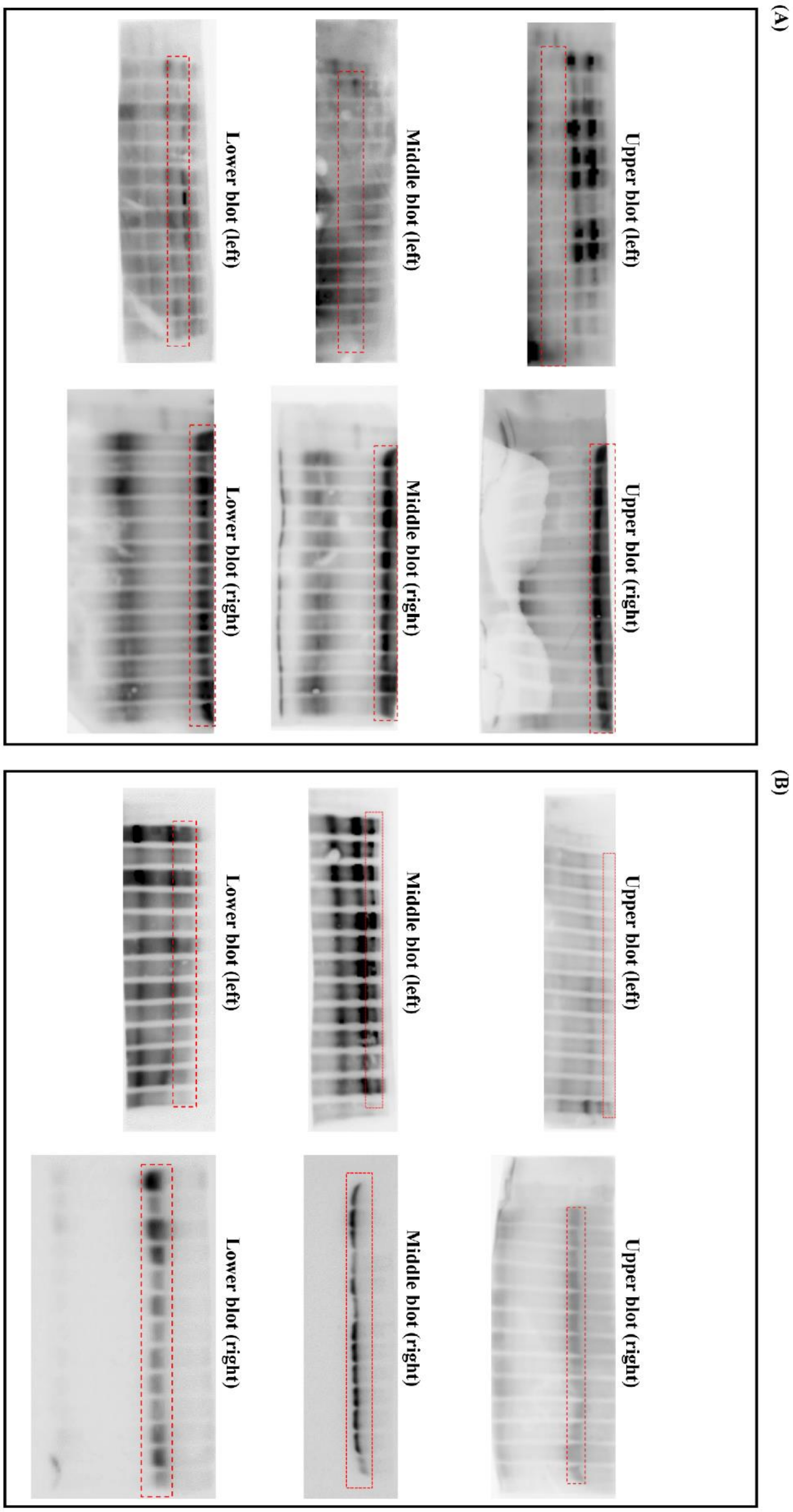

Supplement: Supplementary file 1 — Supplementary Information [file 41598_2017_9691_MOESM1_ESM.pdf]
